# A Design of Copper(II) Coordination Polymers with l‑Homoserine: Structural, Spectroscopic, and Biological Studies

**DOI:** 10.1021/acsomega.5c05776

**Published:** 2025-09-24

**Authors:** Darko Vušak, Ivana Kekez, David Kučera-Čavara, Jurica Jurec, Dijana Žilić, Marta Šimunović Letić, Elena Horvatić, Marija Mioč, Nives Galić, Biserka Prugovečki

**Affiliations:** † Department of Chemistry, Faculty of Science, 117036 University of Zagreb, Horvatovac 102a, Zagreb 10000, Croatia; ‡ Laboratory for Magnetic Resonances, Division of Physical Chemistry, Ruđer Bošković Institute, Bijenička cesta 54, Zagreb 10000, Croatia; § Laboratory of Experimental Therapy, Division of Molecular Medicine, Bijenička cesta 54, Zagreb 10000, Croatia

## Abstract

Five copper­(II) coordination polymers—*trans*-[Cu­(μ-l-hser)­(l-hser)]*_n_* (**1a**), {*cis*-[Cu­(μ-l-hser)_2_]·H_2_O}*_n_* (**1b·H_2_O**), {[Cu­(μ-l-hser)­(H_2_O)­(bpy)]_2_SO_4_·2H_2_O}*_n_* (**2·2H_2_O**), {[Cu­(μ-l-hser)­(H_2_O)­(bpy)]_2_SO_4_·3H_2_O}*_n_* (**2·3H_2_O**), and {[Cu­(μ-l-hser)­(H_2_O)­(bpy)]_2_SO_4_·4H_2_O}*_n_* (**2·4H_2_O**)—were synthesized via solution-based and/or mechanochemical methods (l-hser = l-homoserinate, bpy = 2,2’-bipyridine). The compounds were characterized in the solid state (using single-crystal and powder X-ray diffraction (SCXRD and PXRD), EPR, Raman and IR spectroscopy, and thermogravimetric analysis (TGA)) and in solution (using UV–vis spectroscopy, EPR, Raman spectroscopy, and fluorimetry). In the binary bis­(homoserinato)­copper­(II) compounds, **1a** and **1b·H_2_O**, polymerization is achieved through the hydroxyl group of the l-homoserinate side chain. In contrast, all ternary compounds (**2·2H_2_O**, **2·3H_2_O**, and **2·4H_2_O**) achieved polymerization through the carboxylate group of l-homoserinate. Successful solid-state transformation of **2·2H_2_O** into **2·4H_2_O** was established at higher relative humidity values. Copper­(II) centers adopt a square-pyramidal geometry in **1a** and a distorted octahedral geometry in all other investigated compounds. Spectroscopic studies suggest that **1a** retains its solid-state geometry in solution, while **2·2H_2_O** and **2·3H_2_O** exhibit slight changes. Among the compounds, **2·2H_2_O** exhibited moderate antiproliferative activity against H460 (lung), MCF-7 (breast), and HCT116 (colon) cancer cell lines, and demonstrated antibacterial activity against *Moraxella catarrhalis* ATCC 23246. Absorption and fluorescence spectra suggested a lower binding affinity of **2·2H_2_O** to the double-stranded DNA dodecamer ds­(CGCGAATTCGCG).

## Graphical abstract

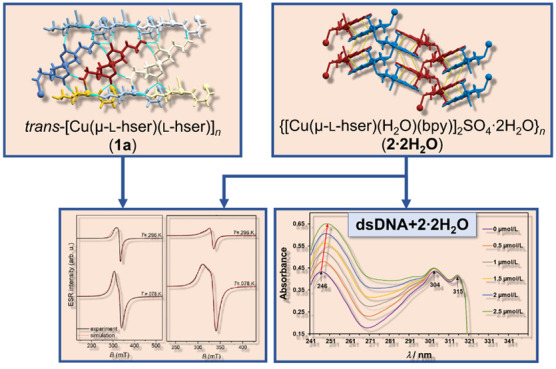

## 1 Introduction

Copper is an essential metal in humans in the oxidized Cu­(II) and reduced Cu­(I) states and is implicated directly or indirectly in the pathogenesis of some human neurological diseases (Alzheimer’s,^1^ Menkes,^2^ Huntington’s,^3^ Parkinson’s,^4^ prion disease,^5^ etc.). It is fundamental to the formation and function of several enzymes and proteins, such as cytochrome C oxidase, catechol oxidase, ascorbate oxidase, Cu/Zn superoxide dismutase, and tyrosinase.^6^ Various copper coordination compounds have been synthesized, structurally characterized, and investigated for their potential therapeutic and diagnostic applications.^7–10^ It is well-known that copper­(II) bis­(aminocarboxylate) compounds are contained in human serum as the physiological species with the dominance of [Cu­(His)_2_], [Cu­(Thr)_2_], and mixed [Cu­(His)­(Ser)] compounds.^11^ Nowadays, binary bis­(aminocarboxylate) copper­(II) compounds with all standard amino acids, except l-cysteine, are synthesized and structurally characterized.^12^ Across all examined crystal structures of copper­(II) complexes with bis­(aminocarboxylate) ligands, the copper­(II) centers exhibit coordination geometries that are either square planar, square pyramidal, or octahedral. Square-pyramidal coordination is found in most *cis*-isomers, where two aminocarboxylates are bound in the equatorial plane, while in most such compounds, a water molecule occupies the apical position.^12^ *Trans*-isomers frequently assemble into coordination polymers, where copper­(II) ions adopt an octahedral geometry. Typically, two aminocarboxylate ligands occupy equatorial sites, while axial positions are commonly bridged by carboxylate groups from adjacent complexes. It is well-known that the bis­(aminocarboxylate)­copper­(II) compound [Cu­(His)_2_] is used for relieving symptoms in Menkes disease, while [Cu­(Gly)_2_] is used for the treatment of skin conditions.^13^ Ternary coordination complexes involving metal ions, amino acids, and heterocyclic bases have been primarily studied due to their significant antitumor properties. The mode of their activity has been correlated to the overproduction of reactive oxygen species (ROS) and their coupling to DNA.^14–20^ Ternary copper­(II) coordination compounds with amino acidates and heterocyclic bases are part of a very extensively researched group of Casiopeina compounds having general formulas [Cu­(N–N)­(α-l-amino acidato)]­NO_3_ and [Cu­(N–N)­(O–O)]­NO_3_, where N–N is an aromatic heterocyclic base (2,2’-bipyridine, 1,10-phenanthroline or their substituted analogues) and O–O is acetylacetonate or salicyaldehydate.^20–22^ Generally, three possible modes of action of the Casiopeinas have been proposed: mitochondrial toxicity, the generation of ROS, or the ability of the compounds to bind and interact with DNA’s nucleic acids through terminal base-pair stacking, minor-groove binding, or intercalation.^23–25^

To date, the structural features of copper­(II) ternary complexes comprising 2,2’-bipyridine and 15 standard amino acids have been structurally characterized. Such structures mainly contain Cu atoms pentacoordinated by an amino acidate ion (via carboxylate oxygen atom and amino nitrogen atom) and 2,2’-bipyridine (via two nitrogen atoms) in equatorial positions and apically coordinated water molecules. In some structures, Cu atoms are octahedrally coordinated with the carboxylate oxygen atoms of neighboring complex ions or other ions (Cl^–^, ClO_4_^–^, NO_3_^–^) occupying the sixth coordination position.^12^

l-Homoserine is a nonessential chiral amino acid that plays a key role in the biosynthetic pathways of other amino acids, including l-threonine and l-methionine.^26^ This study represents only the third reported structural analysis of coordination compounds involving l-homoserine (the first one being the ruthenium complex with l-homoserine,^27^ and the second one copper coordination compounds with 1,10-phenanthroline and l-homoserine.^28^

Our recent research has focused on the synthesis of novel copper­(II) ternary coordination complexes incorporating various amino acids and heterocyclic bases—such as 2,2’-bipyridine and 1,10-phenanthroline—with the aim of developing potential DNA-binding agents exhibiting antiproliferative effects against diverse cancer cell lines.^28–33^ In this paper, we report syntheses and structural investigation of five new copper coordination compounds with l-homoserine (Hhser) and 2,2’-bipyridine (bpy): *trans*-[Cu­(μ- l -hser)­(l-hser)]*_n_* (**1a**), {*cis*-[Cu­(μ-l-hser)_2_]·H_2_O}*_n_* (**1b·H_2_O**), {[Cu­(μ-l-hser)­(H_2_O)­(bpy)]_2_SO_4_·2H_2_O}*n* (**2·2H_2_O**), {[Cu­(μ-l-hser)­(H_2_O)­(bpy)]_2_SO_4_·3H_2_O}*n* (**2·3H_2_O**) and {[Cu­(μ-l-hser)­(H_2_O)­(bpy)]_2_SO_4_·2H_2_O}*_n_* (**2·4H_2_O**). The crystallization behavior of a specific solvate was studied under varying conditions, including solvent type, temperature, and relative humidity. Solid-state characterization was performed using single-crystal and powder X-ray diffraction (SCXRD and PXRD), infrared and Raman spectroscopy, as well as thermal analysis techniques. Additionally, the local magnetic environment of copper­(II) ions was explored through electron paramagnetic resonance (EPR) spectroscopy or electron spin resonance (ESR). Electronic spectral properties of both binary and ternary compounds were analyzed in solution. The biological activity of selected compounds, specifically **1a** and **2·2H_2_O**, was evaluated in terms of antiproliferative and antibacterial effects. The most promising drug candidate, **2·2H_2_O**, was further assayed using UV–vis spectroscopy and fluorimetry to test possible interaction with the ds­(CGCGAATTCGCG) in solution.

## 2 Results and Discussion

### 2.1 Syntheses and Crystallizations

Bis­(homoserinato)­copper­(II) compounds, **1a** and **1b·H_2_O**, were prepared by reaction of copper­(II) hydroxide and l-homoserine in a stoichiometric ratio of 1:2 using water and methanol as solvents, respectively. Compound **1a** crystallized by slow solvent evaporation at room temperature, while **1b·H_2_O** crystallized by cooling a hot solution. The formation of ternary coordination compounds—**2·2H**_**2**_ **O**, **2·3H**_**2**_ **O**, and **2·4H**_**2**_ **O**—was found to be influenced by the water content in the reaction medium. These three solvatomorphs were obtained via solution-based synthesis involving anhydrous or pentahydrate copper­(II) sulfate, copper­(II) hydroxide, l-homoserine, and 2,2’-bipyridine. Compound **2·4H_2_O** crystallized from an aqueous solution and copper­(II) sulfate pentahydrate as a reactant. Compound **2·3H_2_O** crystallized from the methanol solution or a mixture of methanol and water in a volume ratio of 1:1 and using copper­(II) sulfate pentahydrate as a reactant. Compound **2·2H_2_O** was obtained by lowering the water concentration even further and using methanol and anhydrous copper­(II) sulfate (Figure 1).

**Figure 1:**
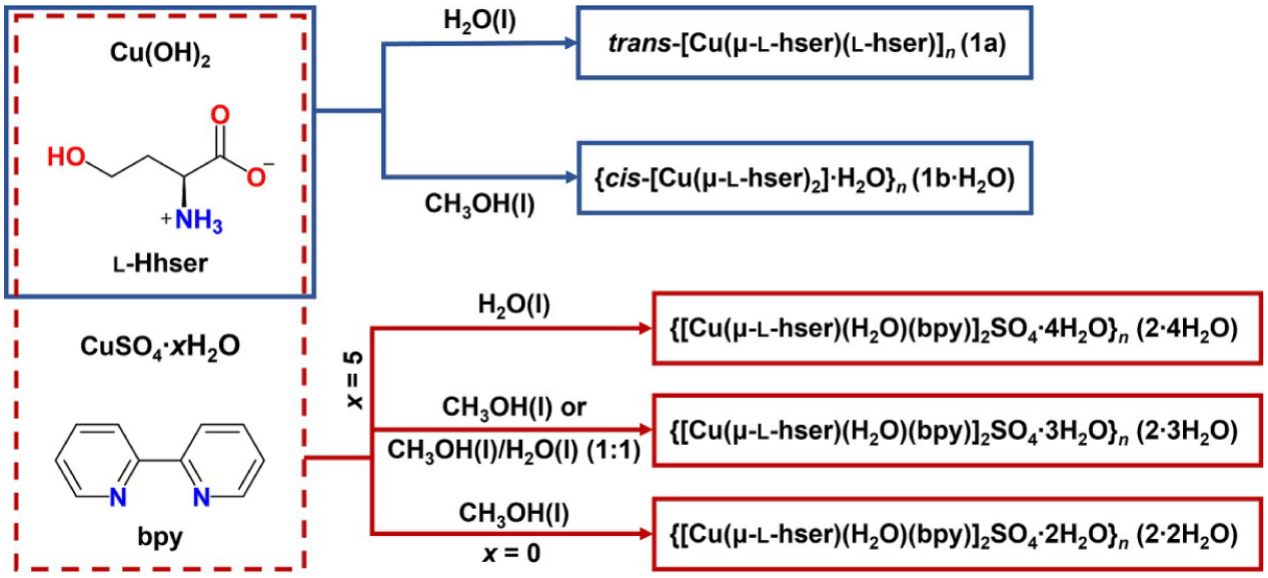
Schematic diagram of solution-based syntheses of **1a**, **1b·H_2_O**, **2·2H_2_O**, **2·3H_2_O**, and **2·4H_2_O**.

A similar procedure was followed for mechanochemical syntheses, where the ratio of water and methanol was adjusted (Figure 2). Only **2·2H_2_O** was prepared purely by liquid-assisted grinding (LAG). We demonstrated that the solvent has a significant influence on the final product of LAG synthesis. If methanol or a mixture of methanol and water in ratios 9:1 or 7:3 (*v*/*v*) was used, pure **2·2H_2_O** was obtained (Figure 2). For a higher fraction of water in the reaction mixture (a mixture of methanol and water 1:1, *v*/*v*, or pure water), **2·2H_2_O** formed in a mixture with unknown phase(s), as observed in the PXRD pattern (Figure 2).

**Figure 2:**
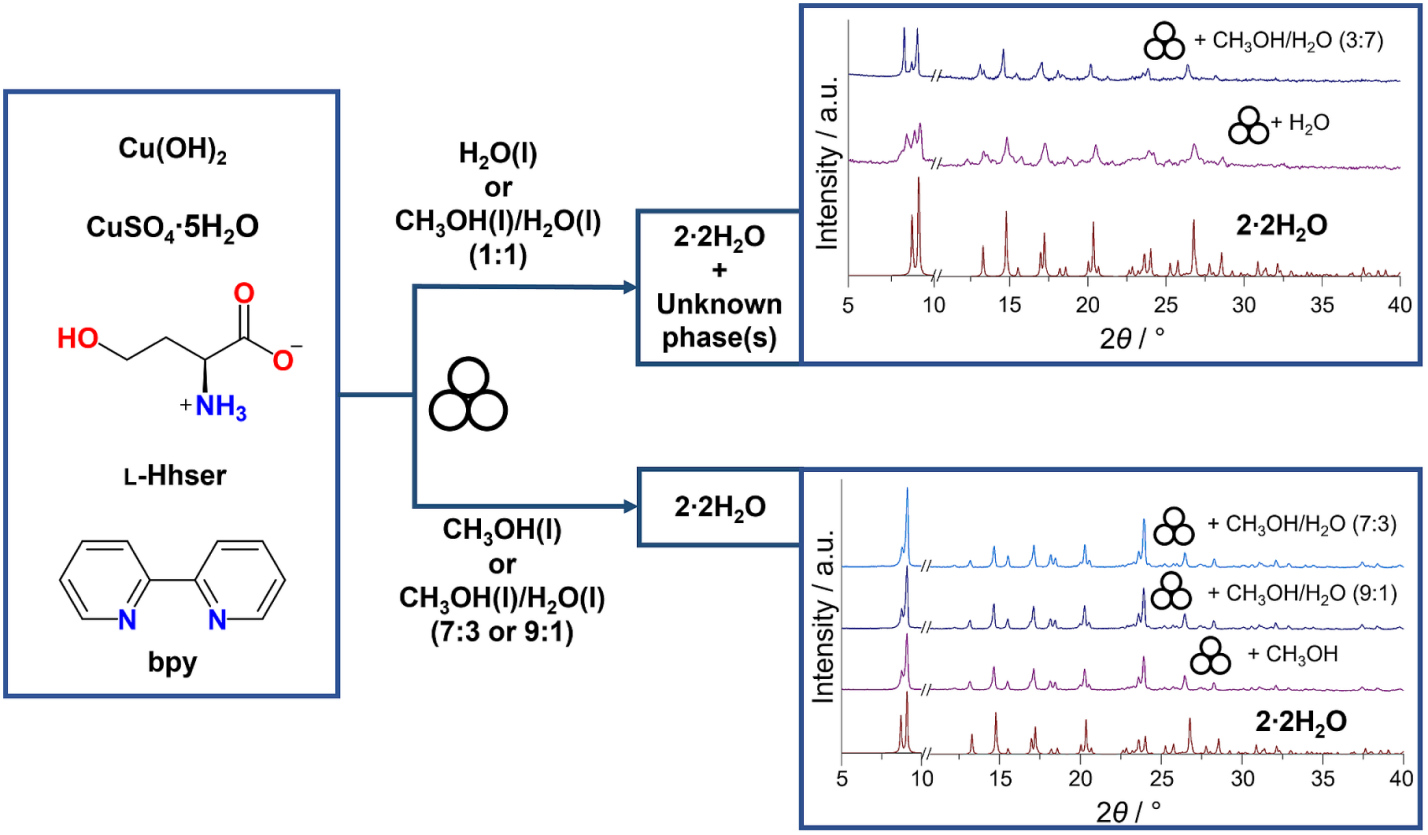
Schematic diagram of LAG syntheses. PXRD patterns calculated from single-crystal structure data of **2·2H_2_O** are shown in red, while experimental PXRD patterns are shown in purple and blue. Parts of the PXRD patterns separated by broken lines are not on the same intensity scale.

### 2.2 Crystal Structures

In bis­(homoserinato)­copper­(II) compounds, **1a** and **1b·H_2_O**, the Cu atom is coordinated by two *N*,*O*-donating homoserinate ligands. In **1a,** they coordinate the copper ion in the equatorial plane to form a *trans,* and in **1b·H_2_O**, they create a *cis-* isomer (Figure S1). In **1a**, one of the l-homoserinate ligands acts as a bridge between two copper atoms and is coordinated to the neighboring copper atom through the hydroxyl group, forming a 1D coordination polymer (Figure 3). The geometry of the five-coordinate copper center in **1a** was evaluated using the τ_5_ descriptor (τ_5_ = 0.13), indicating a predominantly square-pyramidal geometry with minor distortion.^34^ In **1b·H_2_O**, the hydroxyl groups of both l- homoserinate ligands act as a bridge between two copper atoms, completing the distorted octahedral geometry and forming a 2D coordination polymer (Figure 3). Figure S2 depicts an overlay of the molecular structures of **1a** and **1b·H**_**2**_ **O**. Apical Cu–O bonds are elongated due to the Jahn–Teller effect (*d* = 2.394(2) Å in **1a**, and 2.664(5) and 2.690(5) Å in **1b·H_2_O**, Table S1). The average copper–ligand bond length in **1b·H_2_O** is 2.205 Å and ζ parameter is 1.888 Å (average of the sum of the deviation of 6 unique copper-ligand bond lengths around the central metal atom).^35^ In compound **1b·H_2_O**, significant deviations from the ideal octahedral bond angles are evident. The *cis* angles, which ideally measure 90°, range from 83.6(2)° to 99.5(2)°), while the *trans* angles, ideally 180°, are 169.69(16)° and 174.5(2)), as shown in Table S2. The ∑ parameter, which represents the total deviation of 12 unique *cis* ligand-copper-ligand angles from 90°, amounts to 62.14°.^36,37^ Water molecule in the structure **1b·H_2_O** forms hydrogen bonds with ligands, and it is not coordinated to the copper atom, which is less common for this type of compound. This is the first report of *trans*-Cu­(AA)_2_ (AA = amino acidate) and the second report of *cis*-Cu­(AA)_2,_ where polymerization is achieved through the hydroxyl group of the side chain. Several crystal structures of Cu­(AA)_2_ have been reported in CSD involving serine, threonine, tyrosine, and derivatives of tyrosine, 3-hydroxytyrosine, and 3,5-dibromotyrosine. In most cases, compounds are polymers and polymerization is achieved through the carboxylate group, except in the case of {c*is*-[Cu­(tyr-OH)_2_]·H_2_O}*_n_* (tyr-OH = 3-hydroxytyrosine), where complexes are polymerized through the hydroxyl group of the 3-hydroxytyrosinato ligand.^38^ In compound {[Cu­(μ-ser)­(H_2_O)_3_]_2_SO_4_}_*n*_ ^39^ polymerization is also achieved through the hydroxyl group of the serinato ligand.

**Figure 3:**
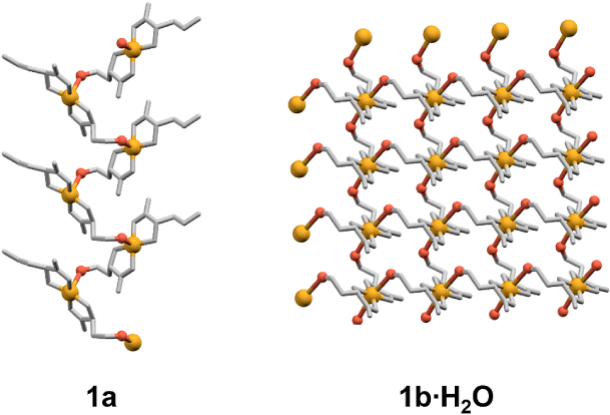
1D polymeric chain in **1a** and 2D layer in **1b·H_2_O**. Hydrogen atoms were omitted for clarity. Copper­(II) atoms are orange, and the bridging oxygen atoms are red, while the rest of the atoms are gray.

In **1a**, polymeric chains propagate along the *b*-axis in a zigzag fashion (Figure 4). Chains are connected to four adjacent polymeric chains through O_hydroxyl_–H···O_carboxylate_ (*d* = 2.812(3)–3.140(3) Å), N–H···O_carboxylate_ (*d* = 2.899(3)–2.994(2) Å) and N–H···O_hydroxyl_ (*d* = 2.899(2) Å) hydrogen bonds (Table S3) forming 3D supramolecular framework. In **1b·H_2_O**, N–H···O_carboxylate_ (*d* = 3.086(7)−3.268(8) Å) and O_hydroxyl_–H···O_carboxylate_ (*d* = 2.707(7) and 2.715(7) Å) hydrogen bonds are present in 2D polymeric layers (Figure 4). Only dispersion interactions are observed between layers.

**Figure 4:**
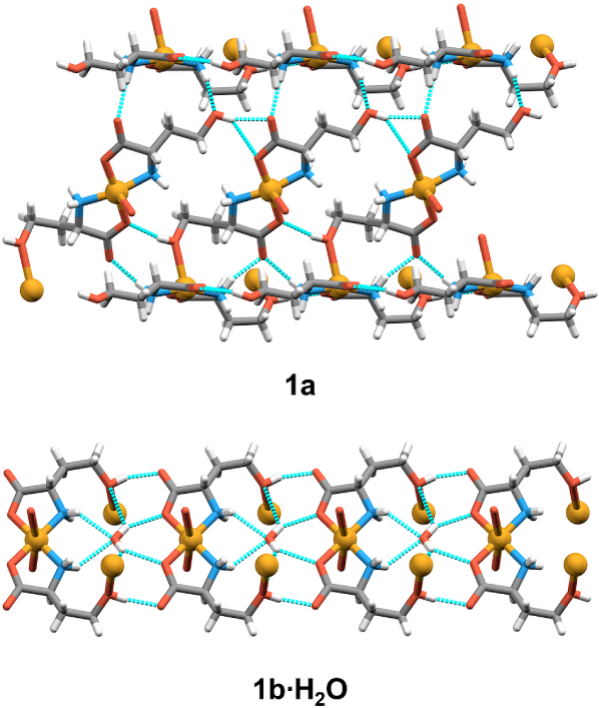
Selected hydrogen bonds shown as cyan dashed lines between complex units in **1a** and **1b·H_2_O**.

The ternary compounds **2·2H**_**2**_ **O**, **2·3H**_**2**_ **O**, and **2·4H**_**2**_ **O** share an identical secondary building unit (SBU), [Cu­(μ-l-hser)­(H_2_O)­(bpy)]^+^. In this unit, the l-homoserinate ligand coordinates via its amino nitrogen and carboxylate oxygen, while the 2,2’-bipyridine ligand binds through its nitrogen atoms, forming chelate rings around the copper­(II) center in the equatorial plane (Figure S3). The SBU also features a water molecule occupying one axial position and a carboxylate group from a neighboring complex in the other, resulting in the formation of a one-dimensional polymeric chain (Figures 5 and 6). Axial Cu–O_water_ (*d* = 2.355(5)–2.631(11) Å) and Cu–O_carboxylate_ (*d* = 2.511(11)–2.804(5) Å) are elongated due to the Jahn–Teller effect (Table S1). In **2·2H_2_O** and **2·4H_2_O**, in all symmetrically independent SBUs, Cu–O_water_ distances are shorter than Cu–Ocarboxylate bonds. In **2·3H_2_O**, two symmetrically independent SBUs contain shorter Cu–O_water_ than Cu–Ocarboxylate bonds, while another two SBUs show opposite lengths. Due to *trans*-influence in octahedral SBUs, most of the axial bond distances in **1b·H_2_O**, **2·2H_2_O**, **2·3H_2_O**, and **2·4H_2_O** are longer than the apical Cu–O bond in **1a** (Table S1), and most of the elongated pentacoordinated copper complexes found in the CSD database (median value of apical Cu–O bond in 74983 data sets is 2.31 Å). In all compounds with 2,2’-bipyridine C and A optical isomers are present, as can be seen in different coordination of axial ligands, however, with different torsion angles of l-homoserine residue (Figure S4). Additionally, **2·2H**_**2**_ **O** and **2·3H**_**2**_ **O** exhibit disorder in l-homoserinato residue as well as sulfate ion. The average copper–ligand bond lengths in compounds **2·2H**_**2**_ **O**, **2·3H**_**2**_ **O**, and **2·4H**_**2**_ **O** range from 2.161 Å to 2.197 Å, while the corresponding ζ values span from 1.524 Å to 1.671 Å. The shortest mean bond length and lowest ζ value occur in one of the independent complex cations present in compound **2·3H**_**2**_ **O**. All investigated ternary compounds showed deviations from the ideal octahedral bond angles. The *cis* angles range between 78.94(17) and 103.4(2)° while the *trans* angles are 167.5(2)° and 177.36(16) (Table S2). The ∑ parameters are 64.08° and 62.12° for compound **2·2H**_**2**_ **O**, 56.8°, 46.1°, 58.7°, and 59.7° for compound **2·3H**_**2**_ **O** and 62.95° and 69.98° for compound **2·4H**_**2**_ **O**. An overlay of the complex cations in **2·2H**_**2**_ **O**, **2·3H**_**2**_ **O**, and **2·4H**_**2**_ **O** is shown in Figure S4.

**Figure 5:**
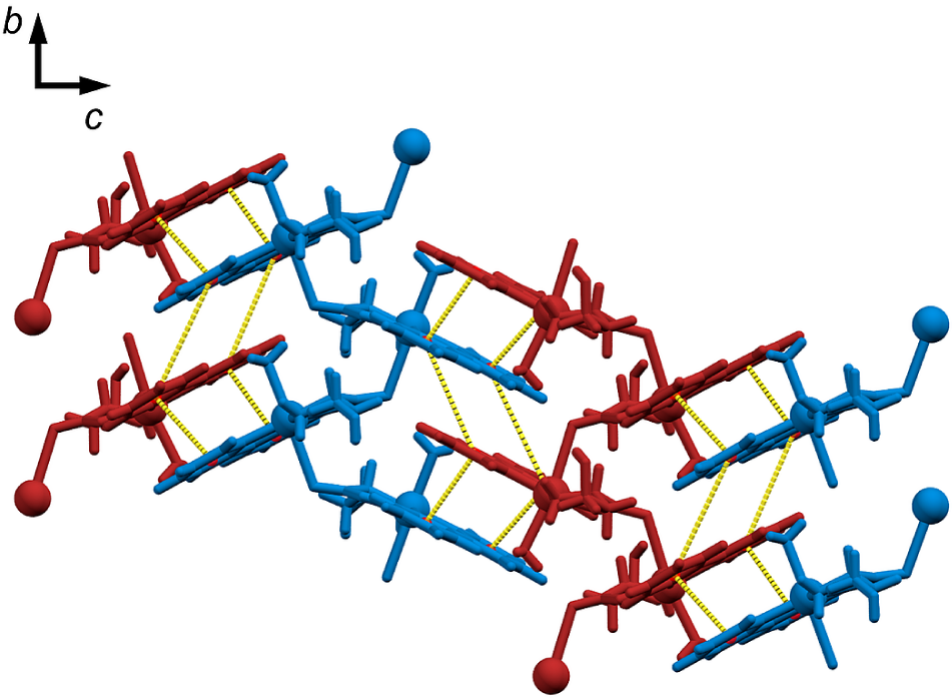
Packing of polymeric chains in **2·4H_2_O** in *b*–*c* crystallographic plane. The shortest contacts of the centroids of the aromatic rings of 2,2’-bipyridine are depicted in yellow dashed lines. Different polymeric chains are shown in blue and red.

**Figure 6:**
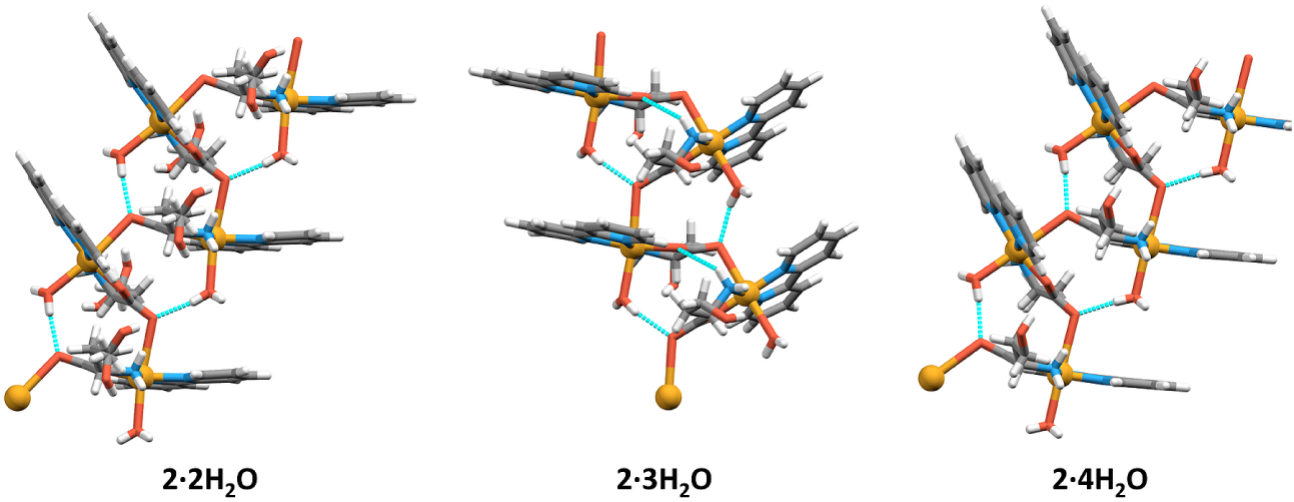
Intramolecular hydrogen bonds (cyan dashed lines) in selected cationic polymeric chains of **2·2H_2_O**, **2·3H_2_O** and **2·4H_2_O**.

In **2·2H_2_O** and **2·4H_2_O**, cationic polymeric chains propagate along the *b*-axis, and in **2·3H_2_O**, chains propagate along the *a*-axis, forming helices. All polymeric chains stack by π-interactions of bipyridine rings, building infinite 2D layers in a zipper-like structure (Figure 5). 2D layers are bridged through hydrogen bonds with sulfate ions and crystallization water molecules into a 3D supramolecular framework. In all three solvates, there is at least one of the following hydrogen bonds: O_water_–H···O_carboxylate_ (*d* = 2.836(13)–2.871 Å), O_water_–H···O_water_ (*d* = 2.690(14)–2.865(7) Å), O_water_–H···O_sulfate_ (*d* = 2.62(3)–2.749(11) Å), O_hydroxyl_–H···O_sulfate_ (*d* = 2.721(19)–2.944(12)­Å), O_hydroxyl_–H···O_water_ (*d* = 2.638(10)–2.906(12) Å), N–H···O_water_ (*d* = 2.892(15)–3.129(8) Å), and N–H···O_sulfate_ (*d* = 2.80(3)–3.197(17) Å) (Figure 6 and Table S3). Additionally, **2·3H_2_O** forms N–H···O_carboxylate_ (*d* = 3.290(15) and 3.359(15) Å) hydrogen bonds. Crystallization water molecules in **2·2H_2_O**, **2·3H_2_O**, and **2·4H_2_O** pack into discrete pockets, occupying 2.5, 6.6 and 8.9%, respectively (Figure S5).

### 2.3 Interconversion **2·2H_2_O** → **2·4H_2_O** in Solid-State

Powder samples of **2·2H_2_O** were placed into atmospheres of different relative humidities (*RH*), and their stability was monitored by PXRD. **2·2H_2_O** remains stable over a wide range of relative humidities from 0–79% even after 60 days. At *RH* = 85% formation of **2·4H_2_O** was observed in PXRD patterns, but the transformation is slow. After 10 days at *RH* = 85%, only a few additional peaks with low intensity were observed. After 60 days, the compound **2·2H_2_O** was almost completely converted into **2·4H_2_O**, but some peaks of **2·2H_2_O** remained. At *RH* = 95 and 100%, it completely converted into **2·4H_2_O** (Figures 7, S6, and S7). After 60 days, the sample aged at *RH* = 100% dissolved in condensed water. Interestingly, the formation of **2·3H_2_O** was not observed, which may be due to slightly different intramolecular hydrogen bonding.

**Figure 7:**
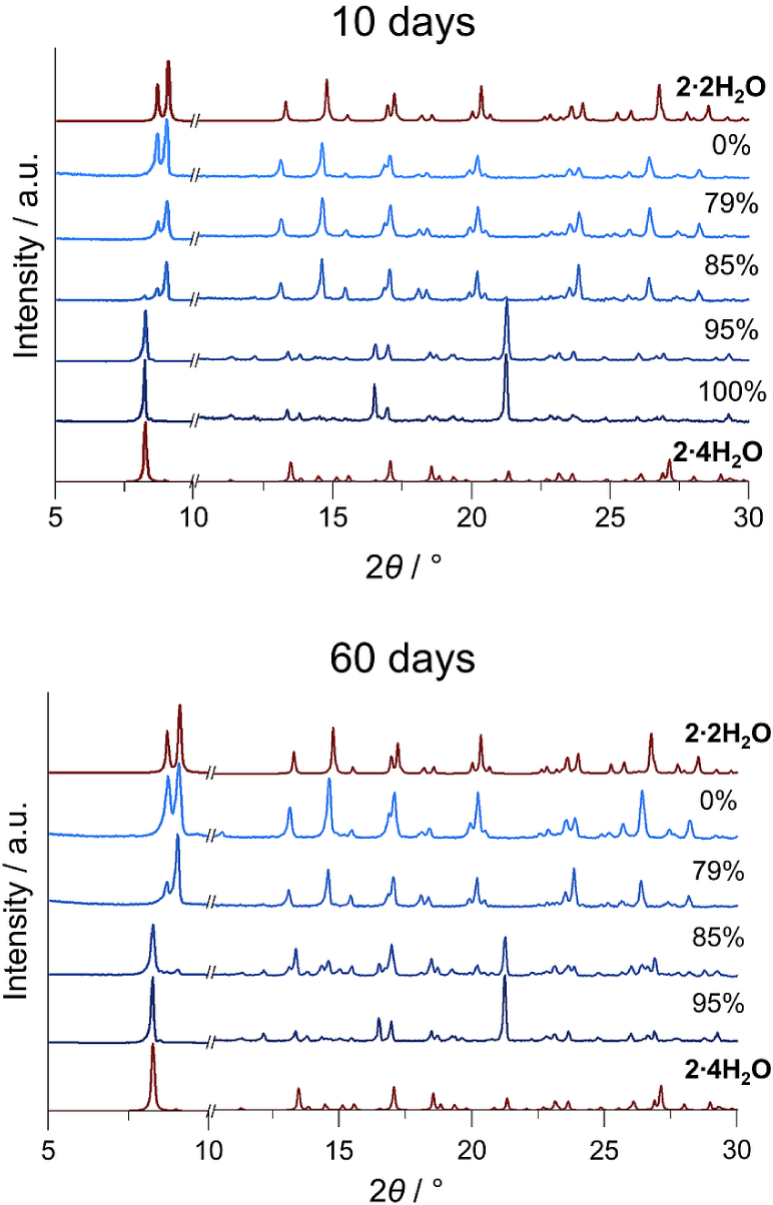
PXRD patterns of the sample of **2·2H_2_O** aged in atmospheres of different relative humidities at 20 °C for 10 and 60 days. PXRD patterns calculated from single-crystal structure data are shown in red, while experimental PXRD patterns are shown in shades of blue. Parts of the diffraction patterns separated by broken lines are not on the same intensity scale.

### 2.4 Thermogravimetric Analysis

Thermogravimetric analysis (TGA) was conducted on compounds that were prepared in pure form and are stable outside of solution, **1a**, **2·2H**_**2**_ **O** and **2·3H**_**2**_ **O**. A summary of the results is presented in Table 1, while the corresponding TGA curves are provided in the (Figure S8). Mass fraction of copper is consistent with the theoretical values, but the water content has a higher error in TG analysis of **2·2H**_**2**_ **O** and **2·3H**_**2**_ **O**. The reason for the deviation is the overlapping of the water loss and the decomposition of the organic part of the complex at approximately 182 °C for **2·2H**_**2**_ **O** and 145 °C for **2·3H**_**2**_ **O**. Compound **1a** decomposes at a higher temperature, 210 °C.

**Table 1:** Summary of the Thermogravimetric Analyses of **1a**, **2·2H**_**2**_ **O**, and **2·3H**_**2**_ **O**

Compound	Temperature of the start of decomposition (temperature of water loss)/°C	*w*(H_2_O, theor.)/%	*w*(H_2_O, exp.)/%	*w*(Cu, theor.)/%	*w*(Cu, exp.)/%
**1a**	210	/	/	21.2	20.8
**2·2H_2_O**	182 (93)	8.5	7.5	15.1	14.6
**2·3H_2_O**	145 (60)	10.5	7.8	14.7	14.0

### 2.5 Infrared and Raman Spectroscopy

Infrared analysis was made in ATR mode for **1a**, **2·2H**_**2**_ **O** and **2·3H**_**2**_ **O** in solid state (Figure S9). IR spectrum of **1a**, **2·2H**_**2**_ **O** and **2·3H**_**2**_ **O** showed typical vibrations for O–H and N–H in the range 3000–3500 cm^–1^ and stretching vibrations of C–H in the region of 2800–3000 cm^–1^. These bands are broad, indicating extensive hydrogen bonding. Broad bands in **2·2H**_**2**_ **O** and **2·3H**_**2**_ **O** in the region 1550–1700 cm^–1^ are assigned to the overlapped vibration modes of the carboxylic group, aromatic C=N and ring stretching vibrations. In **2·2H**_**2**_ **O** and **2·3H**_**2**_ **O**, the highest peak in the region 1550–1700 cm^–1^ is found at 1602 cm^–1^, and in **1a** at 1590 cm^–1,^ confirming the delocalization in the carboxylate group.

In Raman spectra in solid state, most of the higher intensity bands can be assigned to C–H and aromatic ring deformation vibrations. Spectra of **2·2H**_**2**_ **O** and **2·3H**_**2**_ **O** are very similar, with only small deviations of band maxima (Figure S10). Stronger bands assigned to ring deformations occur at 1613, 1579, 1046, and 779 cm^–1^ for **2·2H**_**2**_ **O** and at 1610, 1579, 1043, and 777 cm^–1^ for **2·3H**_**2**_ **O**. Bands assigned to C–H vibrations are found at 1453, 1335, and 1285 cm^–1^ for **2·2H**_**2**_ **O** and at 1449, 1332, and 1281 cm^–1^ for **2·3H**_**2**_ **O**.^40–42^ Raman spectra of the aqueous solutions of **2·2H**_**2**_ **O** and **2·3H**_**2**_ **O** are almost identical, indicating a similar structure of complex species in both solutions (Figure S11). Bands with strong intensity in solid state are preserved in solutions of both **2·2H**_**2**_ **O** and **2·3H**_**2**_ **O** at 1616, 1582, 1046, and 778 cm^–1^ for ring vibrations and at 1452 (for **2·2H**_**2**_ **O**) or 1450 (for **2·3H**_**2**_ **O**), 1332 and 1281 cm^–1^ for C–H vibrations. In **1a** C–H vibrational bands are observed at 1322 cm^–1^ and 1049 cm^–1^, while the C–C vibration appears at 913 cm^–1^ (Figure S10). Raman spectrum of the aqueous solution of **1a** does not show any sharp bands, probably due to the low intensity of bands (Figure S11).

### 2.6 EPR Spectroscopy

Polycrystalline coordination compounds **1a**, **2·2H**_**2**_ **O**, and **2·3H**_**2**_ **O**, along with their DMSO solutions (1 mmol·L^–1^), were examined using X-band EPR spectroscopy. Representative spectra recorded at two selected temperatures (room temperature and liquid nitrogen) are presented in Figure 8. The solution spectra at room temperature were weak and, therefore, were omitted. **1b·H_2_O** was not measured since it crystallizes in a mixture with unknown phases, while **2·4H_2_O** decomposes outside of solution.

**Figure 8:**
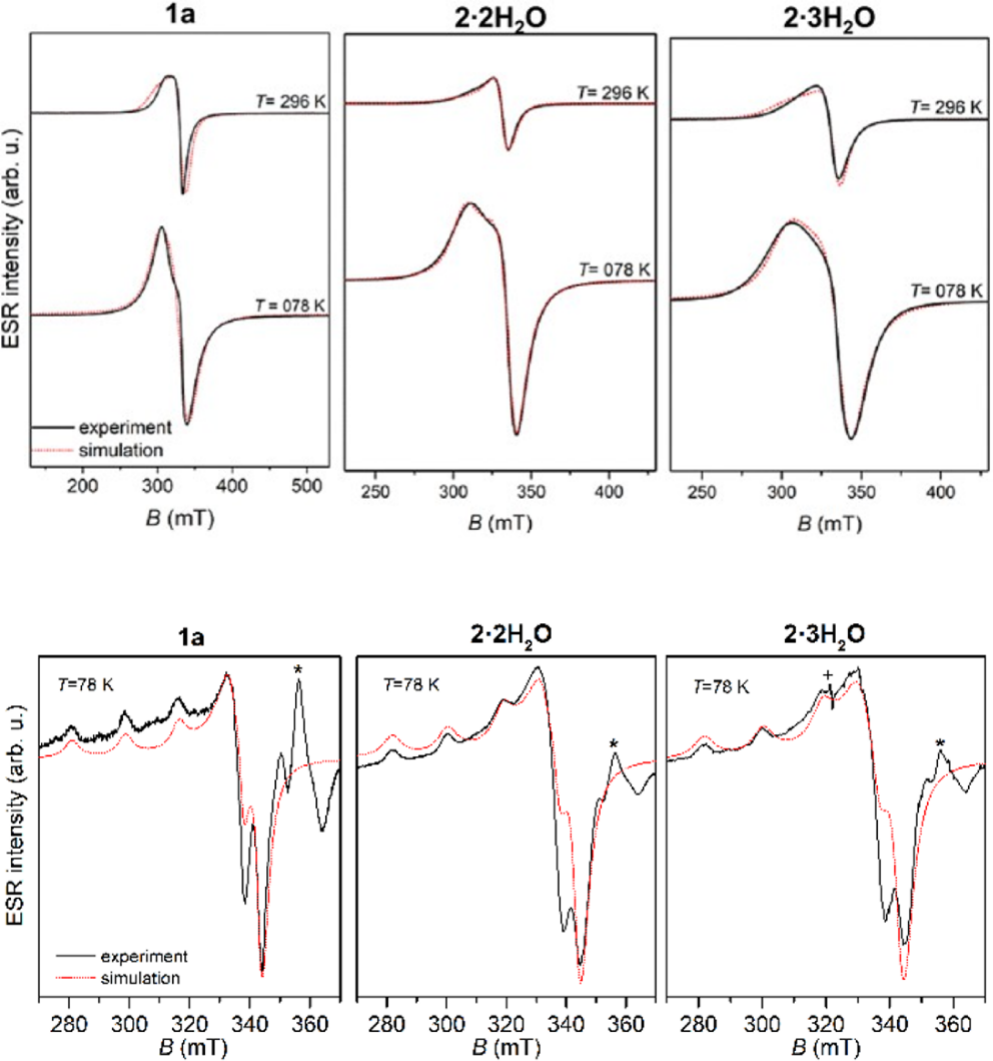
Experimental (black solid lines) and simulated (red dotted lines) EPR spectra of polycrystalline samples (top) and solutions (bottom) of the investigated complexes. The EPR intensities of the polycrystalline spectra at 296 and 78 K are presented in the real ratios. Background signal in the solution spectra is labeled by asterisks, while “+” refers to the signal from the clay used to close the glass capillary.

The spectral simulations were performed by EasySpin software^43^ using the following form of the spin-Hamiltonian for copper­(II) ions (eq 1):^44,45^

1 $$H=\mu_{B}B\cdot g\cdot S+S\cdot A\cdot I$$

where the symbols have their usual meaning.^43–45^ The spin-Hamiltonian parameters derived from spectral simulations are listed in Table 2. For the polycrystalline samples, identical g-tensor values were applied for measurements at both temperatures. The simulations accounted for temperature-dependent variations in line width by adjusting the assumed Lorentzian line shape accordingly. The small variations in the local geometry of Cu­(II) coordination can cause the distribution of *g*_*x*_, *g*_*y*_ and *g*_*z*_-values around some average values.^46^ This effect, described by **g**-strain parameters, is also considered in the simulations of polycrystalline samples with the values given in Table 2.

**Table 2:** Spin-Hamiltonian Parameters Derived from the Spectral Simulations

Complex		**g** = [*g*_*x*_ *g*_*y*_ *g*_*z*_]	**g**-strain	**A** (MHz)	*lw* (mT)	*T* (K)
**1a**	polycryst.	[2.06 2.08 2.26]	[0.00 0.00 0.10]	-	20	296
			[0.00 0.00 0.00]		9	78
	solution	[2.06 2.06 2.25]	-	[50 50 560]	3.4	78
**2·2H**_**2**_ **O**	polycryst.	[2.06 2.06 2.24]	[0.03 0.09 0.31]	-	1	296
			[0.00 0.00 0.05]		11	78
	solution	[2.06 2.06 2.23]	-	[50 50 580]	4.4	78
**2·3H**_**2**_ **O**	polycryst.	[2.05 2.05 2.27]	[0.03 0.01 0.19]	-	8	296
			[0.03 0.00 0.08]		15	78
	solution	[2.06 2.07 2.23]	-	[50 50 580]	4.6	78

The second hyperfine term in eq 1 that describes the interaction between the copper electron spin **S** and the nuclear spin **I** is not visible in polycrystalline spectra. The unresolved hyperfine interaction is the consequence of spin–spin interaction between copper ions, separated by a distance of 6.3666(4) Å in **1a**, 5.8266(12) and 6.0031(12) Å in **2·2H**_**2**_ **O** and 5.526(10) and 5.527(10) Å **2·3H**_**2**_ **O** in polycrystalline samples. By enlarging the distance between copper ions with the solvent molecules, the hyperfine interaction becomes well resolved in solution spectra.

The obtained *g*-values *g_x_* ≈ *g_y_* < *g_z_*, as can be seen in Table 2, are expected for the elongated octahedral, square pyramidal or square planar copper geometry.^32,47^ The obtained EPR values are in agreement with their crystal structures; square-pyramidal geometry in **1a** and elongated octahedral in **2·2H_2_O** and **2·3H_2_O**. The results indicated that the unpaired electron in these compounds is predominantly localized in the $d_{x^{2}-y^{2}}$ orbital.

In solution, the copper geometry is similar to that in polycrystalline samples, as indicated by the comparison of *g*-values in Table 2. However, the solution spectra could not be satisfactorily simulated using the polycrystalline *g*-parameters, which reveal a small but noticeable difference in copper geometry upon dissolution.

### 2.7 Electronic Spectral Analysis of Binary and Ternary Cu­(II) Compounds in Solution

At room temperature, UV–Vis spectra of the binary compound **1a** and the ternary compounds **2·2H**_**2**_ **O** and **2·3H**_**2**_ **O** were recorded in the range 200–900 nm using a 10 mmol L^–1^ Tris-base buffer at pH 7.4 (Table S4). All three compounds exhibit distinct spectral features, indicating their stability in aqueous solution under the experimental conditions.

For compound **1a**, a d–d transition is observed at 628 nm (ε = 66 M^–1^ cm^–1^), while a ligand-to-metal charge transfer (CT) band appears at 263 nm (ε = 3314 M^–1^ cm^–1^). The λ_max_ at 628 nm supports the presence of a pentacoordinated geometry in solution, aligning with previously reported data for structurally related bis-chelated copper­(II)–amino acid complexes.^48^ In comparison, **2·2H**_**2**_ **O** exhibits a d–d transition band at 607 nm (ε = 82 M^–1^ cm^–1^), along with three charge-transfer (CT) bands at 256, 304, and 316 nm, each displaying markedly higher molar absorptivities. These CT bands suggest enhanced ligand-to-metal charge transfer, likely from the σ-donor nitrogen atoms of bipyridine to the Cu­(II) $d_{x^{2}-y^{2}}$ orbital.^49^ The spectral pattern of **2·2H**_**2**_ **O** is consistent with either a square pyramidal or an octahedral geometry in solution. This conclusion is supported by both the experimental UV–Vis data and literature reports demonstrating that ternary Cu­(II) complexes with similar donor sets can adopt both geometries depending on solvent effects.^50–53^ The UV–Vis spectrum of **2·3H**_**2**_ **O** closely resembles that of **2·2H**_**2**_ **O** (Table S4), indicating that variations in crystal hydration do not significantly affect the solution-state coordination geometry or electronic structure.

### 2.8 Biological Activity of **1a** and **2·2H_2_O**

To assess how ligand coordination influences biological activity, compounds **1a** and **2·2H_2_O** were selected for further evaluation. The proliferation assay revealed that **1a** exhibited no detectable activity against any of the three tested cell lines: HCT116, MCF-7, and H460. Contrary to bis­(homoserinato)­copper­(II) compound **1a**, ternary compound **2·2H_2_O** incorporating 2,2’-bipyridine exhibited moderate activities (*IC*_50_) toward the tested cell lines in a range of 15.8–19.3 μmol dm^–3^ (Table 3). Previously reported ternary coordination compounds, {[Cu­(μ-l-Ala)­(H_2_O)­(bipy)]_2_SO_4_·2H_2_O}*_n_* and {[Cu­(μ-l-Val)­(H_2_O)­(bipy)]­[Cu­(l-Val)­(H_2_O)­(bipy)]_3_(SO_4_)_2_·4H_2_O}*_n_*, demonstrated notable antiproliferative activity against human hepatocellular carcinoma cell lines (HepG2), along with moderate efficacy against human acute monocytic leukemia cell lines (THP-1).^30^ Similarly, as in case of **2·2H_2_O**, already published data for α-[Cu­(l-Ser)­(H_2_O)­(bpy)]_2_SO_4_ showed moderate antiproliferative activity toward three cell lines, HCT116 (colon carcinoma), H460 (lung carcinoma) and MCF-7 (breast carcinoma) and in the range of 10–18 μmol dm^–3^.^29^ All these results suggest that the replacement of polar amino acid homoserine with nonpolar amino acids (alanine, valine) or polar amino acid containing a shorter hydrocarbon side chain length (serine) does not significantly affect the antiproliferative activity of ternary Cu­(II) compounds. On the contrary, the replacement of heterocyclic base from 2,2’-bipyridine to 1,10–phenanthroline increases the antitumor activity approximately 10 times (Table 3) as observed in the case of {[Cu­(μ-l-hser)­(H_2_O)­(phen)]­[Cu­(μ-l-hser)­(phen)]­SO_4_·6H_2_O}*_n_*.^28^ Comparing the antiproliferative activity among the other compounds of similar structural properties already tested,^33^ it is clear that by modifying the heterocyclic base, it is possible to tune this class of compounds for a possible application as anticancer drugs.

**Table 3:** *IC*_50_ Values of **1a** and **2·2H_2_O**, Compared to {[Cu­(μ-l-hser)­(H_2_O)­(phen)]­[Cu­(μ-l-hser)­(phen)]­SO_4_·6H_2_O}*_n_*, Etoposide and 5-Fluorouracil (in μM)^28,54^

*IC*_50_ ^a^/10^–6^ mol dm^–3^			
	Cell lines		
Compound	HCT116	MCF-7	H 460
**1a**	≥100	≥100	≥100
**2·2H_2_O**	15.8 ± 0.5	18.6 ± 0.25	19.3 ± 0.6
{[Cu(μ-l-hser)(H_2_O)(phen)][Cu(μ-l- hser)(phen)]SO_4_·6H_2_O}_*n*_ ^b^	1.5 ± 0.3	1.7 ± 0.02	2.13 ± 0.17
etoposide	5 ± 2^c^	1 ± 0.7^c^	0.1 ± 0.04^c^
5-fluorouracil	4 ± 1^c^	14 ± 0.3^c^	3 ± 0.3^c^

a *IC*_50_—the concentration that causes 50% growth inhibition.

b Ref. 28.

c Ref. 54.

Recently, El-Sayed et al.^55^ showed that two ternary Cu­(II) complexes ([Cu­(HPf)­(bipy)­(NO_3_)]­NO_3_·2H_2_O and [Cu­(HPf)­(phen)­(NO_3_)]­NO_3_·2H_2_O) (HPf = pefloxacin) possess antibacterial activity against *Escherichia coli* that is even greater than that of Gentamicin, a commonly used standard antibiotic. To evaluate the antibacterial potential of our compounds, an in vitro assay was conducted on **2·2H**_**2**_ **O** and **1a** against four pathogenic microorganisms: two Gram-positive bacteria: *Staphylococcus aureus* ATCC29213 and *Enterococcus faecalis* ATCC29212 and two Gram-negative bacteria: *Escherichia coli* TolC-Tn10 and *Moraxella catarrhalis* ATCC23246 (Table 4). Compound **2·2H_2_O** showed moderate activity (MIC) of 64 μg/mL only toward the Gram-negative *M. catarrhalis* ATCC 23246 strain. However, among the four tested bacterial strains, no activity was found for compound **1a**, which lacks the coordinated heterocyclic base (Table 4).

**Table 4:** MIC Values of **2·2H_2_O** and **1a** Compared to Azithromycin and Ciprofloxacin (in μg/mL)

MIC^a^ (μg/mL)				
Compound	*S. aureus* ATCC 29213	*E. faecalis* ATCC29212	*M. catarrhalis* ATCC 23246	*E. coli* TolC-Tn10
**2·2H_2_O**	>256	>256	64	>256
**1a**	>256	>256	>256	>256
azithromycin	0.5	4	≤0.06	0.25
ciprofloxacin	0.25	0.25	≤0.06	0.25

a MIC—minimal inhibitory concentration of an antimicrobial drug that will inhibit the visible growth of a microorganism after overnight incubation.

*M. catarrhalis* is a Gram-negative human bacterial pathogen of the respiratory tract and is commonly associated with nasopharynx otitis in children or infections of the lower respiratory tract, whereas in adults it causes acute exacerbations of chronic obstructive pulmonary disease. Since the compound **2·2H_2_O** shows moderate antibacterial activity against *M. catarrhalis* this class of ternary Cu­(II) complexes could be used for future design of more potent antibacterial drugs.

The interactions between transition metal complexes and DNA/RNA have been extensively studied and are the focus of many investigations, including the development of new chemotherapeutic drugs. Understanding the binding modes of these complexes to DNA provides valuable insights into the biochemical mechanisms underlying their action. We performed absorption titration spectroscopy to test the binding affinity of **2·2H_2_O** to ds­(CGCGAATTCGCG) (dsDNA). The UV spectrum of **2·2H_2_O** buffered aqueous solution without dsDNA contained three absorption maxima at 246, 304, and 315 nm. (Figure 9). The addition of dsDNA increased the absorption intensity at 246 nm (hyperchromic effect) and slightly shifted toward the longer wavelengths (bathochromic shift, Δλ = 3 nm) (Figure 9). The same hyperchromic effect was observed for [Cu­(phen)­(l-Thr)­(H_2_O)]­ClO_4_ ternary complex^56^ as well as other copper­(II) complexes with a ligand bearing an −OH group,^57^ suggesting a possibility for either groove or intercalating binding mode of this class of complexes.^58^ Additionally, since the ternary complexes carry a strong positive charge, they could bind electrostatically to the negatively charged structures of DNA and RNA. They can also form hydrogen bonds between the polar groups of amino acids and the nitrogen bases of nucleotides. To estimate the binding constant (*K*_b_), the Wolfe-Shimmer equation was applied (inset of Figure 9) using the absorption at 246 nm and the *K*_b_ of 6.37 × 10^3^ M^–1^ was calculated. According to the data obtained, the value of *K*_b_ is equal to the binding constant of [Cu­(phen)­(l-Thr)­(H_2_O)]­ClO_4_ to the calf thymus DNA (CT-DNA),^56^ while 3 orders of magnitude lower than that of classical intercalators like ethidium bromide (*K*_b_ = 1.4 × 10^6^ M^–1^).^59^ In addition, the binding of **2·2H_2_O** to dsDNA was also investigated using fluorescence spectroscopy, performing a competitive DNA-binding assay between **2·2H_2_O** and GelRed dye (GR).^60^ The results, illustrated in Figure 10, indicated that the fluorescence intensity of the GR–dsDNA complex decreased by adding the **2·2H_2_O**. A small quenching of the emission band of the GR–dsDNA complex with the addition of the complex **2·2H_2_O** implies that the **2·2H_2_O** complex weakly competes for DNA-binding sites with GR (the calculated Stern–Volmer binding constant was *K*_SV_ = 1.64 × 10^3^ M^–1^, Figure 10). All these findings suggest that the binding of 2·2H**_2_** O to dsDNA is partial intercalation. Since the calculated binding constant of 6.37 × 10^3^ M^–1^ is quite small for intercalation and huge for electrostatic binding, it could correspond to an external interaction. This interaction probably occurs between the functional component of the ternary complex (heterocyclic base) and the major or minor grooves of a double-stranded DNA.

**Figure 9:**
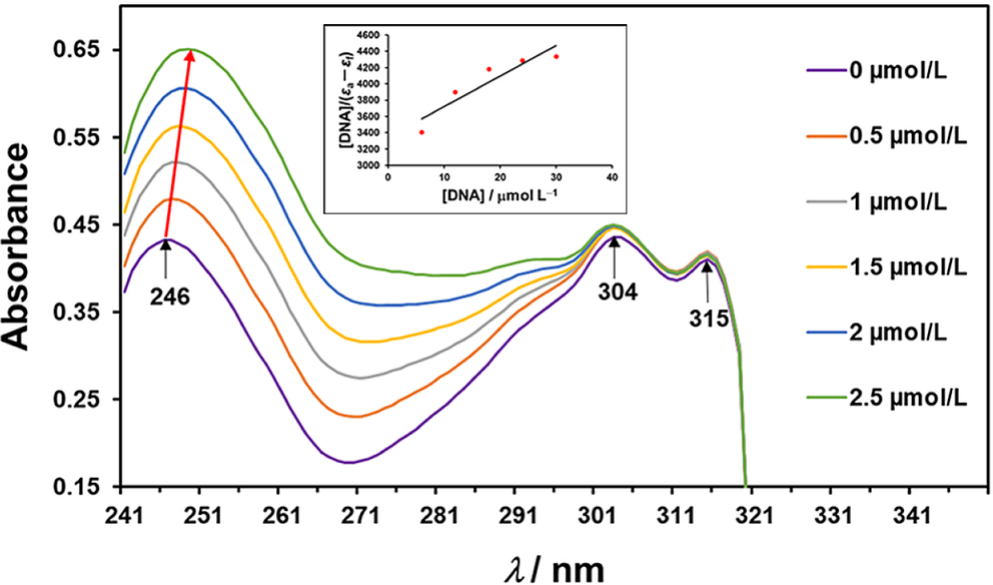
UV spectrum of 30 μmol L^–1^ **2·2H_2_O** with increasing concentrations of dsDNA (from 0 to 2.5 μmol L^–1^). Black arrows indicate three bands of **2·2H_2_O** absorption without added dsDNA. Absorbance increases with the addition of dsDNA and slightly shifts toward the longer wavelengths (highlighted with a red arrow). The Wolfe–Shimmer plot, drawn using the change in absorbance at 246 nm, is inserted.

**Figure 10:**
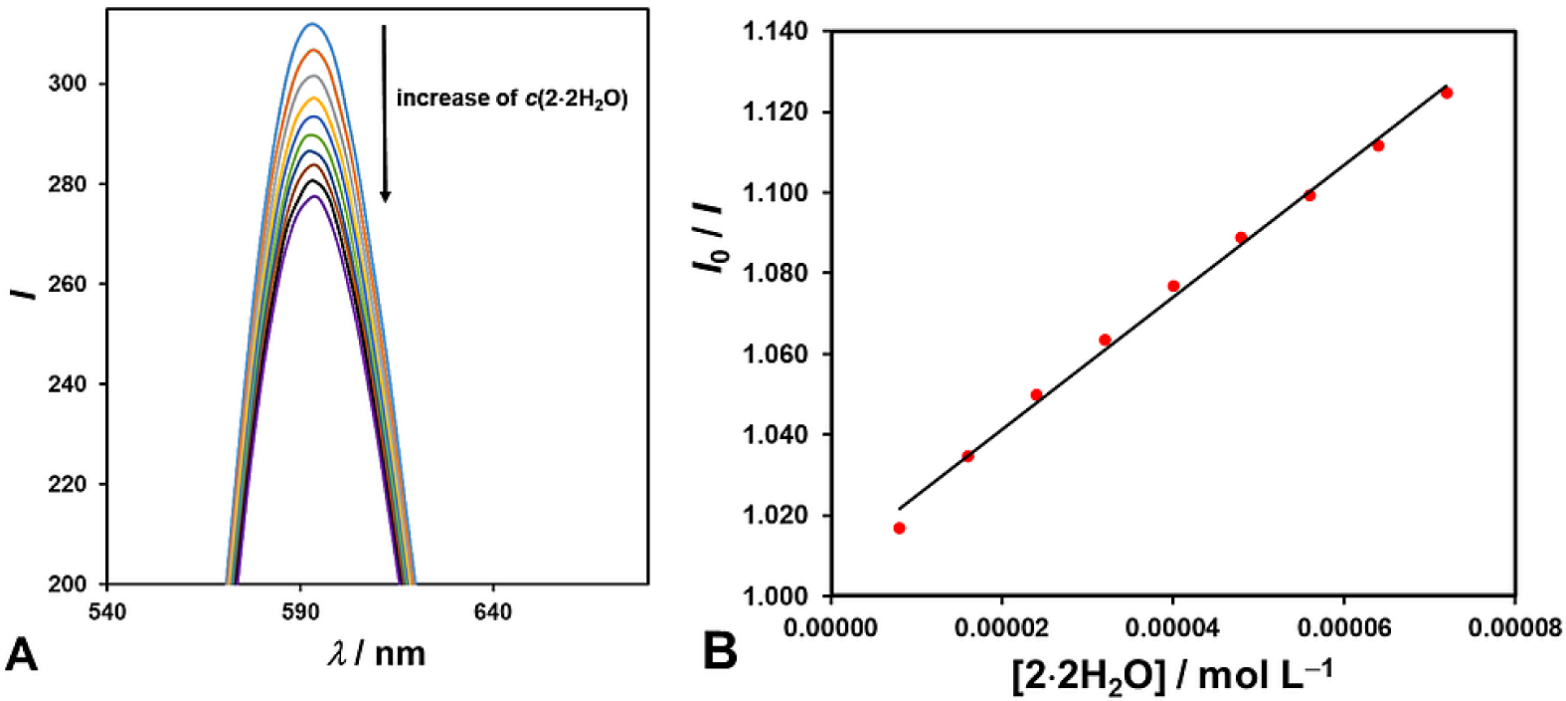
(A) Emission spectra of 2 μmol L^–1^ GR–dsDNA in the absence (top blue curve) and presence of the **2·2H_2_O** complex with increasing concentrations (0, 8, 16, 24, 32, 40, 48, 56, 64, and 72 μmol L^–1^). The arrow indicates the emission intensity changes upon increasing the concentration of **2·2H_2_O**. (B.) The Stern–Volmer plot of *I*_0_/*I* vs [**2·2H_2_O**] was drawn using the change in fluorescence intensities at 593 nm.

## 3 Conclusion

In this study, we synthesized (by solution-based methods) and structurally characterized five copper­(II) coordination polymers with l-homoserine, both in binary forms (**1a** and **1b·H_2_O**) and ternary forms incorporating 2,2’-bipyridine (**2·2H_2_O**, **2·3H_2_O**, and **2·4H_2_O**). **2·2H_2_O** was synthesized by mechanochemical synthesis using four reactants in solid state and with a specific solvent used for LAG (methanol or methanol/water mixtures in ratio 9:1 or 7:3). The polymerization mode varied, with **1a** (1D chains) and **1b·H_2_O** (2D layers) polymerizing via the hydroxyl group and the ternary compounds **2·2H_2_O**, **2·3H_2_O**, and **2·4H_2_O** all forming 1D chains via the carboxylate group. Stability experiments of **2·2H_2_O** in an atmosphere of different relative humidities (*RH*) revealed that **2·2H_2_O** is stable from 0 to 79% and it transforms into **2·4H_2_O** at higher *RH*. EPR spectroscopy revealed that the unpaired copper electron in **1a**, **2·2H_2_O**, and **2·3H_2_O** compounds is localized in the $d_{x^{2}-y^{2}}$ orbital. The EPR spectra with unresolved hyperfine interaction indicate spin–spin interaction between copper atoms. UV–Vis and EPR spectra confirmed that compound **1a** retains its solid-state geometry in solution, whereas **2·2H_2_O** and **2·3H**_2_ **O** exhibited minor differences, likely due to solvent effects. Biological investigations showed that **2·2H_2_O** possesses moderate antiproliferative activity against three human cancer cell lines and selective antibacterial activity against *M. catarrhalis*. In contrast, the binary complex **1a** was inactive, underscoring the crucial role of the coordinated heterocyclic ligand in modulating biological activity. Spectroscopic DNA-binding studies suggest that **2·2H_2_O** interacts externally with the grooves of double-stranded DNA. These findings highlight the importance of ligand design in tuning the structure and bioactivity of copper­(II) coordination polymers. This work lays the groundwork for the future development of copper-based systems with enhanced therapeutic potential, particularly in the search for new antibacterial and anticancer agents.

## 4 Materials and Methods

All reagents for syntheses and experiments (copper­(II) sulfate pentahydrate, Gram- mol, Zagreb, Croatia; copper­(II) hydroxide, Alfa Aesar, Ward Hill, USA; l-homoserine, Fluorochem, Hadfield, UK; 2,2’-bipyridine, Acros Organics, Geel, Belgium; phosphorus­(V) oxide, Acros Organics, Geel, Belgium; sodium hydroxide, Carlo Erba Reagents, Cornaredo, Italy; potassium acetate, Kemika, Zagreb, Croatia; magnesium chloride hexahydrate, Kemika, Zagreb, Croatia; potassium carbonate, Fischer Chemicals, Zurich, Switzerland; magnesium nitrate hexahydrate, Merck, Rahway, USA; cobalt­(II) chloride, Kemika, Zagreb, Croatia; sodium chloride, Alkaloid, Skopje, North Macedonia; ammonium chloride, Kemika, Zagreb, Croatia; potassium chloride, Kemika, Zagreb, Croatia; potassium nitrate, Alkaloid, Skopje, North Macedonia; methanol, Carlo Erba Reagents, Cornaredo, Italy; nitric acid, Kemika, Zagreb, Croatia; tris­(hydroxymethyl)­aminomethane (Tris-base buffer), Sigma-Aldrich, St. Louis, USA; double-stranded oligonucleotide (ds­(CGCGAATTCGCG), Metabion service; and GelRed dye (GR), Merck) were obtained from commercial sources and used without purification. Anhydrous copper­(II) sulfate was prepared by heating copper­(II) sulfate pentahydrate to 220 °C, followed by cooling to room temperature under a dry atmosphere. Its purity was verified using powder X-ray diffraction analysis.

Powder X-ray diffraction (PXRD) data were measured on a PANalytical Aeris diffractometer in the Bragg–Brentano geometry using Cu*K*_α_ radiation (λ = 1.54056 Å) at room temperature. PXRD data were collected in a 2θ range of 5–40° with a step of 0.022° and 15.045 s per step.

Mechanochemical syntheses were performed using a Retsch MM200 ball mill in Teflon jars (*V* = 14 mL) with one stainless steel ball (diameter 8 mm) and at a vibration frequency of 25 Hz.

UV spectra were recorded using Analytik Jena Specord 200 Spectrometer in the range of 240–320 nm, with a slit width of 2 nm. Fluorescence spectra were recorded on the PerkinElmer LS55 Spectrometer using slit widths of 10 nm for excitation and emission.

EPR (electron paramagnetic resonance) or ESR (electron spin resonance) measurements were conducted using a Bruker Elexsys 580 FT/CW spectrometer over a temperature range spanning from room temperature to liquid nitrogen temperature. The experiments employed a microwave frequency of approximately 9.7 GHz, with a magnetic field modulation amplitude of 0.5 mT and a modulation frequency of 100 kHz. The spectra of the solutions (*c* = 1 mM in DMSO) were measured using a narrow glass capillary sealed with clay and placed in a quartz tube.

Raman spectra were recorded using a Renishaw inVia Raman microscope with 785 nm laser excitation. A × 5 objective lens (NA = 0.12) was employed for all measurements. Solid samples were placed in aluminum holders, while liquid samples were contained in aluminum pans (volume: 40 μL; outer diameter: 5.4 mm; height: 2.6 mm). Measurement parameters, including laser power and exposure time, were adjusted according to sample type: solutions were analyzed at 160 mW laser power, whereas solid samples were measured at 16 mW. Each sample was illuminated for 10 seconds during acquisition. Raw spectra were processed using WiRE 5.3 software.

The infrared (IR) spectra were acquired in attenuated total reflectance (ATR) mode using a Thermo Scientific Nicolet iS50 FTIR spectrometer, in the spectral range of 4000–400 cm^–1^. Thermogravimetric analysis (TGA) was performed with a Mettler-Toledo TGA/DSC 3+ instrument under an oxygen flow of 50 mL min^–1^, applying a heating rate of 10 °C min^–1^ across a temperature range of 25–800 °C. Samples weighing approximately 5–8 mg were placed in standard alumina crucibles with a volume of 70 μL.

### 4.1 Solution-Based Syntheses

In general, solution-based syntheses of **1a** and **1b·H_2_O** were performed using copper­(II) hydroxide and l-homoserine in a molar ratio of 1:2 (Figure 1), with water or methanol as solvents. The ternary compounds **2·2H**_**2**_ **O**, **2·3H**_**2**_ **O**, and **2·4H**_**2**_ **O** were synthesized by reacting copper­(II) hydroxide, copper­(II) sulfate pentahydrate, l-homoserine, and bipyridine in a 1:1:2:2 molar ratio. The reactions were carried out using water, methanol, or a water–methanol mixture as solvents (Figure 1).

#### 4.1.1 Synthesis of *trans*-[Cu­(μ-l-hser)­(l-hser)]*_n_* (**1a**)

Copper­(II) hydroxide (48.8 mg; 0.5 mmol) and l-homoserine (119.2 mg; 1.0 mmol) were placed in a beaker with 10 mL of water and heated at a boiling temperature for 30 min. The resulting solution was filtered and left to evaporate slowly at room temperature. After a few weeks, large blue crystals of **1a** formed, suitable for single-crystal X-ray diffraction. Crystals of **1a** are stable outside of the solution.

IR (ATR) ν̃/cm^–1^: 3395 (m), 3291 (s), 3236 (s), 3139 (m), 2955 (m), 2914 (m), 2876 (w), 2852 (w), 1608 (s), 1572 (s), 1486 (w), 1466 (w), 1436 (w), 1370 (s), 1333 (s), 1308 (m), 1270 (w), 1230 (w), 1197 (w), 1144 (s), 1105 (m), 1059 (s), 1028 (s), 957 (m), 907 (m), 895 (m), 827 (m), 783 (m), 732 (m), 667 (m), 622 (s), 597 (s), 506 (w), 484 (w), 436 (m), 403 (m).

Raman (solid state) ν̃/cm^–1^: 1611 (m), 1472 (m), 1405 (w), 1349 (m), 1322 (m), 1166 (w), 1045 (m), 986 (w), 960 (w), 913 (m), 789 (w), 739 (w), 655 (w), 589 (m), 509 (m), 445 (w), 397 (w), 334 (w), 275 (w), 225 (s).

#### 4.1.2 Synthesis of {*cis*-[Cu­(μ-l-hser)_2_]·H_2_O}*_n_* (**1b·H_2_O**)

Copper­(II) hydroxide (48.8 mg; 0.5 mmol) and l-homoserine (119.2 mg; 1.0 mmol) were placed in a beaker with 10 mL of methanol and heated at a boiling temperature for 30 min. The resulting solution was filtered and evaporated to a third of the starting volume at an elevated temperature. Blue crystals of **1b·H_2_O** formed upon cooling, suitable for single-crystal X-ray diffraction. Unknown impurities crystallize with **1b·H_2_O**, and we were not able to purify **1b·H_2_O** nor determine the structure of the impurities. Crystals of **1b·H_2_O** are stable outside of the solution.

#### 4.1.3 Synthesis of {[Cu­(μ-l-hser)­(H_2_O)­(bpy)]_2_SO_4_·2H_2_O}*_n_* (**2·2H_2_O**)

Copper­(II) hydroxide (24.4 mg; 0.25 mmol), anhydrous copper­(II) sulfate (62.4 mg; 0.25 mmol), l-

homoserine (59.5 mg; 0.5 mmol) and 2,2’-bipyridine (78.1 mg; 0.5 mmol) were placed in a beaker with 10 mL of methanol and heated at a boiling temperature for 30 min. The resulting solution was filtered and left to evaporate at room temperature. After several days, small blue crystals of **2·2H_2_O** formed, suitable for single-crystal X-ray diffraction. Crystals of **2·2H_2_O** are stable outside of the solution.

IR (ATR) ν̃/cm^–1^: 3307 (s), 3119 (s), 3065 (s), 2907 (m), 2844 (s), 1626 (m), 1602 (s), 1501 (m), 1479 (m), 1446 (m), 1404 (m), 1368 (w), 1321 (m), 1258 (w), 1204 (w), 1158 (m), 1088 (s), 1060 (s), 1050 (s), 1035 (s), 975 (m), 957 (w), 895 (m), 813 (w), 771 (m), 732 (m), 663 (w), 652 (w), 640 (w), 604 (m), 557 (m), 551 (m), 484 (m), 473 (m), 419 (m).

Raman (solid state) ν̃/cm^–1^: 1613 (s), 1579 (m), 1510 (m), 1453 (w), 1355 (s), 1285 (m), 1265 (w), 1246 (w), 1225 (w), 1195 (w), 1178 (w), 1118 (w), 1076 (m), 1046 (s), 985 (m), 909 (w), 816 (w), 779 (m), 657 (w), 569 (w), 483 (w), 383 (m), 334 (w), 254 (m).

Raman (aqueous solution) ν̃/cm^–1^: 1616 (s), 1582 (m), 1510 (m), 1452 (w), 1332 (s), 1281 (m), 1172 (w), 1124 (w), 1072 (w), 1046 (s), 990 (m), 927 (w), 778 (m), 672 (w), 664 (w), 573 (w), 481 (w), 377 (m), 326 (w), 259 (m).

#### 4.1.4 Synthesis of {[Cu­(μ-l-hser)­(H_2_O)­(bpy)]_2_SO_4_·3H_2_O}*_n_* (**2·3H_2_O**)

Copper­(II) hydroxide (24.4 mg; 0.25 mmol), copper­(II) sulfate pentahydrate (62.4 mg; 0.25 mmol), l-homoserine (59.5 mg; 0.5 mmol) and 2,2’-bipyridine (78.1 mg; 0.5 mmol) were placed in a beaker with 10 mL of methanol or 10 mL of mixture of water and methanol (1:1 *v*/*v*) and heated at a boiling temperature for 30 min. The resulting solution was filtered and left to evaporate at room temperature. After several days, small blue crystals of **2·3H_2_O** formed, suitable for single-crystal X-ray diffraction. Crystals of **2·3H_2_O** are stable outside of the solution for a few days or weeks.

IR (ATR) ν̃/cm^–1^: 3310 (s), 3281 (s), 3236 (s), 3116 (s), 3067 (s), 3036 (s), 2960 (s), 2940 (m), 2865 (s), 1602 (s), 1498 (m), 1477 (m), 1444 (m), 1405 (m), 1376 (w), 1321 (m), 1258 (w), 1222 (w), 1192 (w), 1157 (m), 1126 (w), 1083 (m), 1050 (s), 1035 (s), 975 (m), 913 (m), 891 (m), 819 (m), 769 (s), 732 (m), 664 (m), 651 (m), 641 (w), 609 (m), 549 (m), 480 (m), 422 (m).

Raman (solid state) ν̃/cm^–1^: 2970 (w), 2960 (w), 2942 (w), 1637 (w), 1610 (s), 1579 (m), 1507 (m), 1476 (m), 1449 (m), 1411 (w), 1372 (m), 1332 (s), 1320 (s), 1281 (m), 1210 (w), 1187 (w), 1098 (w), 1068 (w), 1043 (s), 1005 (w), 985 (w), 917 (m), 892 (m), 829 (m), 777 (m), 674 (w), 558 (m), 492 (w), 380 (w), 368 (w), 336 (w), 253 (m), 212 (m).

Raman (aqueous solution) ν̃/cm^–1^: 1616 (s), 1582 (m), 1510 (m), 1450 (w), 1332 (s), 1281 (m), 1172 (w), 1124 (w), 1072 (w), 1046 (s), 990 (m), 920 (w), 778 (m), 664 (w), 573 (w), 380 (m), 326 (w), 256 (m).

#### 4.1.5 Synthesis of {[Cu­(μ-l-hser)­(H_2_O)­(bpy)]­2SO_4_·4H_2_O}*_n_* (**2·4H_2_O**)

Copper­(II) hydroxide (24.4 mg; 0.25 mmol), copper­(II) sulfate pentahydrate (62.4 mg; 0.25 mmol), l-

homoserine (59.5 mg; 0.5 mmol) and 2,2’-bipyridine (78.1 mg; 0.5 mmol) were placed in a beaker with 10 mL of water and heated at a boiling temperature for 30 min. The resulting solution was filtered and left to evaporate at room temperature. After several days, small blue crystals of 2·4H**_2_** O formed, suitable for single-crystal X-ray diffraction. Crystals of 2·4H**_2_** O decompose outside of the solution.

### 4.2 Mechanochemical Syntheses

#### 4.2.1 General Procedure

Copper­(II) hydroxide (0.25 mmol), copper­(II) sulfate pentahydrate (0.25 mmol), l-homoserine (0.5 mmol) and 2,2’-bipyridine (0.5 mmol) were placed in a Teflon milling jar (volume 14 mL) with one stainless-steel ball (diameter 8 mm). Water, methanol or a mixture of water and methanol in different ratios (1:1, 3:7 and 1:9, *v*/*v*) were added for liquid-assisted grinding (η = 0.2 μL mg^–1^). Milling was performed for 15 min. The resulting powder was characterized by the powder X-ray diffraction (PXRD) (Figure 2). When methanol or water–methanol mixtures (3:7 and 1:9, *v*/*v*) were employed as solvents, pure **2·2H**_**2**_ **O** was obtained. Other solvates were not obtained in a pure form by this method. Detailed synthetic conditions for mechanochemical syntheses are given in Tables S1–S4.

### 4.3 Interconversion **2·2H_2_O** → **2·4H_2_O** in Solid-State

A few milligrams of **2·2H_2_O** was placed in Eppendorf tubes, which were then put into a closed containers containing P_4_O_10_ (*RH* ≈ 0%), water (*RH* = 100%) or a saturated aqueous solution to maintain particular values of relative humidities: sodium hydroxide (*RH* = 9%), potassium acetate (*RH* = 23%), magnesium chloride hexahydrate (*RH* = 33%), potassium carbonate (*RH* = 43%), magnesium nitrate hexahydrate (*RH* = 54%), cobalt­(II) chloride hexahydrate (*RH* = 65%), sodium chloride (*RH* = 75%), ammonium chloride (*RH* = 79%), potassium chloride (*RH* = 85%) and potassium nitrate (*RH* = 95%).^61^ Samples were kept in a laboratory at a constant temperature of 20 °C and analyzed after 10 and 60 days.

### 4.4 Single-Crystal X-ray Crystallography

Single-crystal X-ray diffraction data for crystals of **1a** and **2·4H_2_O** were collected on an Oxford XCalibur Sapphire 3 diffractometer using Mo*K*α radiation (λ = 0.71054 Å) at 150 K. Single-crystal X-ray diffraction data for samples of **1b·H**_**2**_ **O** and **2·3H**_**2**_ **O** were collected at room temperature using a Rigaku XtaLAB Synergy-S diffractometer equipped with a HyPix 6000HE detector and Cu*K*_α_ radiation (λ = 1.54056 Å). Single-crystal X-ray diffraction data for crystals of **2·2H_2_O** were collected on a synchrotron facility Elettra, Trieste, on XRD1 beamline (λ = 0.70000 Å) at 100 K. Data collection and data processing for **1a**, **1b·H_2_O**, **2·3H_2_O** and **2·4H_2_O** were performed using CrysAlisPro software^62^ and for **2·2H_2_O** using iMosflm^63^ incorporated within CCP4 program package.^64^ The crystal structures were solved using SHELXS^65^ and refined with the SHELXL^66^ program, both implemented within the WinGX software suite.^67^ Crystal structures were visualized by Mercury^68^ and ToposPro^69^ programs. Calculation of geometrical parameters was performed by PLATON.^70^ All non-hydrogen atoms, except several carbon atoms in **2·3H_2_O**, were refined anisotropically. Most hydrogen atoms were located in the Fourier difference map and subsequently placed in geometrically calculated positions based on their corresponding functional groups. For water molecules in **1b·H**_**2**_ **O**, **2·2H**_**2**_ **O**, **2·3H**_**2**_ **O**, and **2·4H**_**2**_ **O**, hydrogen atoms were identified in the Fourier difference map and their positions were restrained to an O–H bond length of 0.85(1) Å and an H···H separation of 1.39(2) Å. Sulfate ions in **2·2H**_**2**_ **O** and **2·3H**_**2**_ **O** exhibited positional disorder over two sites, with refined occupancy ratios of 0.43251:0.56749 and 0.78832:0.21168, respectively. Side chains of some symmetrically independent l-homoserinate ligands were disordered over two positions with occupancies refined to values 0.42391:0.57609 and 0.67943:0.32057 in **2·2H_2_O** and 0.55776:0.44224 in **2·3H_2_O**. The total occupancy factor for all disordered atoms was constrained to 1. Crystallographic data for **1a**, **1b·H_2_O**, **2·2H_2_O**, **2·3H_2_O**, and **2·4H_2_O** are given in Table S5.

### 4.5 Proliferation Assays of **1a** and **2·2H_2_O**

Antiproliferative assays were conducted on three human cancer cell lines: H460 (lung carcinoma), MCF-7 (breast carcinoma), and HCT116 (colon carcinoma), following previously established protocols.^54,71^ The compounds **1a** and **2·2H**_**2**_ **O** were evaluated for their cytotoxic effects. All cell lines were cultured as monolayers in Dulbecco’s Modified Eagle Medium (DMEM) supplemented with 10% fetal bovine serum (FBS), 2 mM l-glutamine, 100 U/mL penicillin, and 100 μg/mL streptomycin. Cultures were maintained at 37 °C in a humidified atmosphere containing 5% CO_2_. The MTT test, as described in the Supporting Information in the section Proliferation assays, MTT test, was used to measure the percentage of cell growth. As a result, *IC*_50_ values were calculated for each cell line and tested compound.

### 4.6 In Vitro Antibacterial Activity of **1a** and **2·2H_2_O**

Minimum inhibitory concentrations (MICs) for compounds **1a** and **2·2H_2_O** were determined by the broth microdilution method according to guidelines of the Clinical Laboratory Standards Institute.^72^ Antibacterial susceptibility was determined against two Gram-positive and two Gram-negative bacterial strains. Double dilutions of tested compounds in 96-well microtiter plates were prepared in a 128–0.125 μg/mL concentration range. Compound **2·2H_2_O** was dissolved in DMSO. *Escherichia coli* (ECM1556) and *Staphylococcus aureus* (ATCC29213) were grown on Mueller-Hinton agar plates (by Becton Dickinson, USA), and *Enterococcus faecalis* (ATCC29212) and *Moraxella catarrhalis* (ATCC23246) were grown on Mueller–Hinton agar supplemented with 5% sheep blood. *E. coli* strain ECM1556 is hypersensitive due to efflux pump deficiency (TolC-Tn10). Inocula were prepared using the direct colony suspension method, and each well was inoculated with 5 × 10^4^ CFU. Minimum inhibitory concentration (MIC) values were determined by visual inspection following 20–22 hours of incubation at 37 °C under ambient atmospheric conditions. As a control, samples of azithromycin and ciprofloxacin antibiotics were used to test the antibacterial susceptibility.

### 4.7 DNA Interaction Studies with **2·2H_2_O**

The UV absorbance at 260 and 280 nm of the double-stranded DNA dodecamer ds­(CGCGAATTCGCG) (dsDNA) solution in 10 mmol L^–1^ Tris-base buffer (pH 7.4 adjusted with HNO_3_) gives a ratio of ca. 1.64, indicating that the DNA was sufficiently free of protein.^73^ The DNA concentration was determined by measuring the UV absorption at 260 nm, taking the molar absorption coefficient (ε_260_) of dsDNA as 199349 L mol^–1^ cm^–1^ (Figure S12). Absorption titration measurements were done by varying the concentration of dsDNA while keeping the concentration of **2·2H_2_O** constant (30 μmol L^–1^) in 10 mmol L^–1^ Tris-base buffer (pH 7.4) at room temperature (Table S6). Samples were kept for 5 min for equilibrium before recording each spectrum. Before adding the titrant, the spectrum of **2·2H_2_O** was recorded in the buffer. All spectra were corrected for dilution. The intrinsic binding constant *K*_b_ for the interaction of the studied compound **2·2H_2_O** with dsDNA was calculated by absorption spectral titration data using the following equation (eq 2).^74^

2 $$\left[\mathrm{DNA}\right]/\left(\varepsilon_{a}-\varepsilon_{f}\right)=\left[\mathrm{DNA}\right]/\left(\varepsilon_{b}-\varepsilon_{f}\right)+1/K_{b}\left(\varepsilon_{b}-\varepsilon_{f}\right)$$

ε_a_, ε_f_, and ε_b_ correspond to *A*_obsd_/[**2·2H_2_O**], the extinction coefficient for the free **2·2H_2_O**, and the extinction coefficient for the **2·2H_2_O**–dsDNA complex, respectively. In the plot of [DNA]/(ε_a_ – ε_f_) vs [DNA], *K*_b_ is then given by the ratio of the slope to the intercept.

The fluorescence emission spectra were recorded in the 540–750 nm wavelength range by exciting the GelRed–dsDNA (GR–dsDNA) system at 520 nm at room temperature. The emission spectra of GR bound to DNA in the absence and presence of complexes have been recorded. The experiment was carried out by titrating **2·2H_2_O** (10 × 10^–3^ mol L^–1^ in 10 mmol L^–1^ Tris-base buffer, pH 7.4 adjusted with HNO_3_) into samples containing 2.0 × 10^–6^ mol L^–1^ of dsDNA and 2.0 × 10^–6^ mol L^–1^ of GR. Samples were kept for 5 min for equilibrium before recording each spectrum. All spectra were corrected for dilution. The quenching constant (*K*_SV_) was calculated according to the Stern–Volmer equation (eq 3).^75^

3 $$I_{0}/I=1+K_{SV}\left[Q\right]$$

*I*_0_ and *I* are the fluorescence intensities in the absence and presence of the quencher, respectively. The *K*_SV_ is the quenching constant, and [Q] is the concentration of the quencher. *K*_SV_ is calculated by the slope of this plot.

## Supplementary Material



## Notes

Additional figures and tables, including ORTEPs of all crystal structures, overlays of the structures, crystal packing, PXRD patterns, TGA curves, IR (ATR), Raman and UV–vis spectra, crystallographic data, distances and valence angles within the polyhedra of copper coordination spheres, additional details about proliferation assay experiments, and the UV titration experiments (PDF)

## Notes

CCDC 2465082–2465086 contain the supplementary crystallographic data for this paper. These data can be obtained free of charge via https://www.ccdc.cam.ac.uk/structures/ (accessed on 23 August 2025) or from the CCDC, 12 Union Road, Cambridge CB2 1EZ, UK; Fax: + 44 1223 336033; E-mail: deposit@ccdc.cam.ac.uk.
