# Supplementary material for: A Design of Copper(II) Coordination Polymers with l‑Homoserine: Structural, Spectroscopic, and Biological Studies
Source: ACS Omega. 2025 Sep 24;10(39):45571–84. doi: 10.1021/acsomega.5c05776 (PMC12508938; doi:10.1021/acsomega.5c05776)
Supplement: Supplementary file 1 [file ao5c05776_si_002.pdf]

**A design of copper(II) coordination polymers with L-homoserine: structural, spectroscopic, and biological studies**

Darko Vušak,[1] Ivana Kekez,[1] David Kučera-Čavara,[1] Jurica Jurec,[2] Dijana Žilić,[2] Marta Šimunović Letić,[1] Elena Horvatić,[1] Marija Mioč,[3] Nives Galić[1] and Biserka Prugovečki[1,*]

[1] Department of Chemistry, Faculty of Science, University of Zagreb, Horvatovac 102a
[2] Laboratory for Magnetic Resonances, Division of Physical Chemistry, Ruđer Bošković Institute, Bijenička cesta 54, Zagreb, Croatia
[3] Laboratory of Experimental Therapy, Division of Molecular Medicine, Ruđer Bošković Institute, Bijenička cesta 54, 10000 Zagreb, Croatia

*Corresponding author

e-mail: biserka@chem.pmf.hr

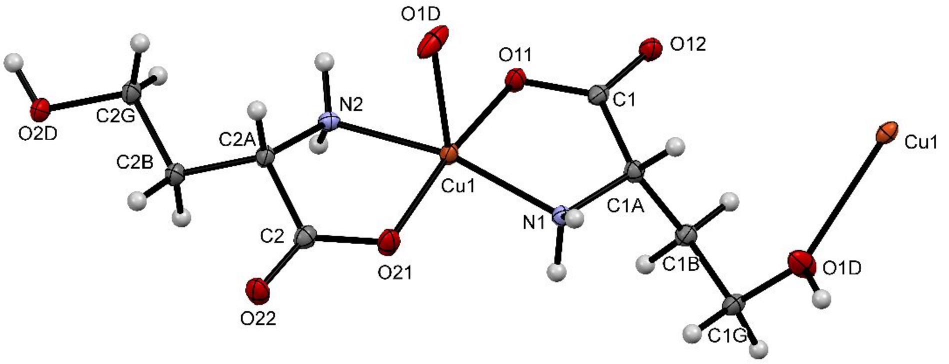


**1a**

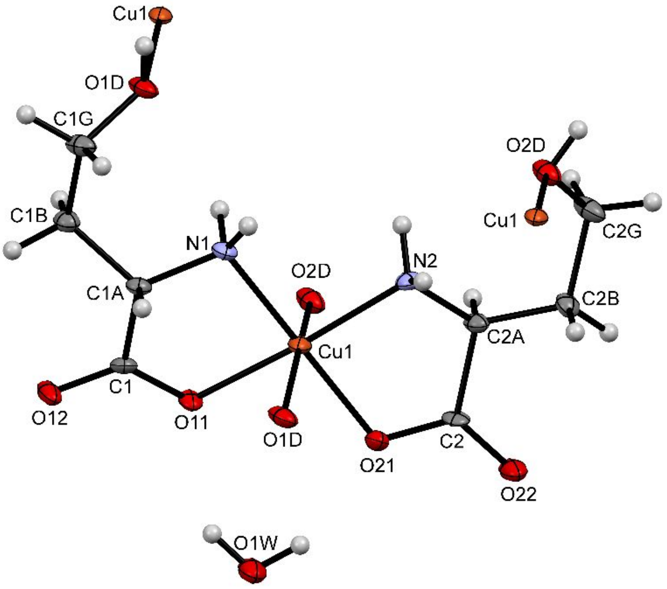


**1b·H$_2$O**

**Figure S1.** Numbering scheme for the coordination polymer *trans*-[Cu(μ-L-hser)(L-hser)]$_n$, **1a**, and {*cis*-[Cu(μ-L-hser)$_2$]·H$_2$O}$_n$, **1b·H$_2$O** (L-hser = L-homoserinate).

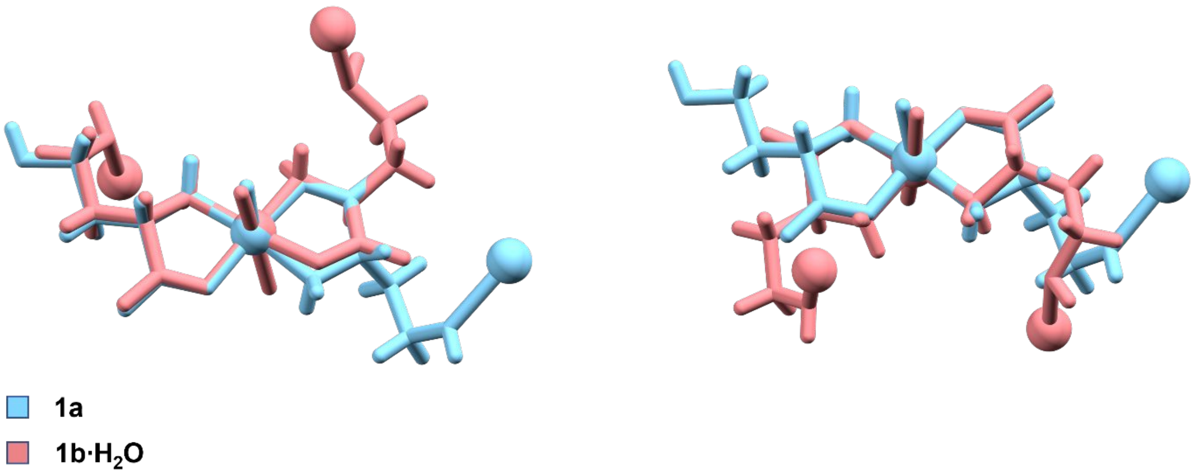


**Figure S2.** Overlay of the structures of **1a** (blue) and **1b·$H_2O$** (red) aligned through the copper centers, one coordinated carboxylate oxygen, and one coordinated nitrogen atom of the L-homoserinate ligands. The crystallization water molecule in **1b·$H_2O$** is omitted for clarity.

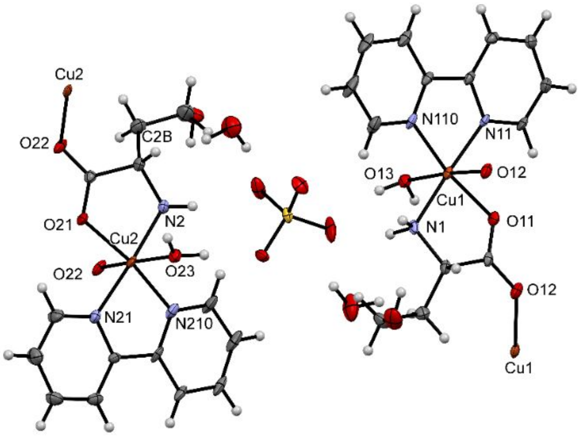


**2·2H$_2$O**

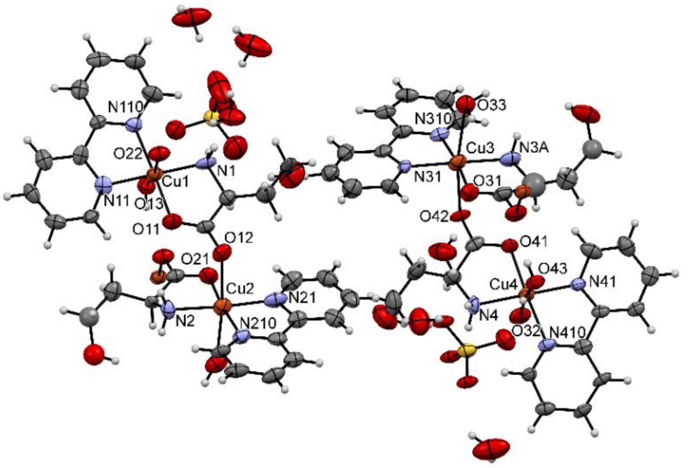


**2·3H$_2$O**

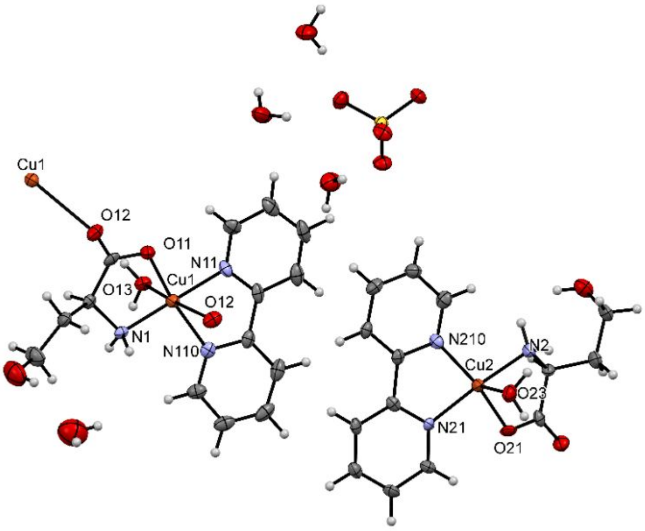


**2·4H$_2$O**

**Figure S3.** Numbering scheme for the coordination polymer {[Cu(μ-L-hser)(H$_2$O)(bpy)]$_2$SO$_4$·2H$_2$O}$_n$, **2·2H$_2$O**, {[Cu(μ-L-hser)(H$_2$O)(bpy)]$_2$SO$_4$·3H$_2$O}$_n$, **2·3H$_2$O**, and {[Cu(μ-L-hser)(H$_2$O)(bpy)]$_2$SO$_4$·4H$_2$O}$_n$, **2·4H$_2$O**, (L-hser = L-homoserinate, bpy = 2,2'-bipyridine).

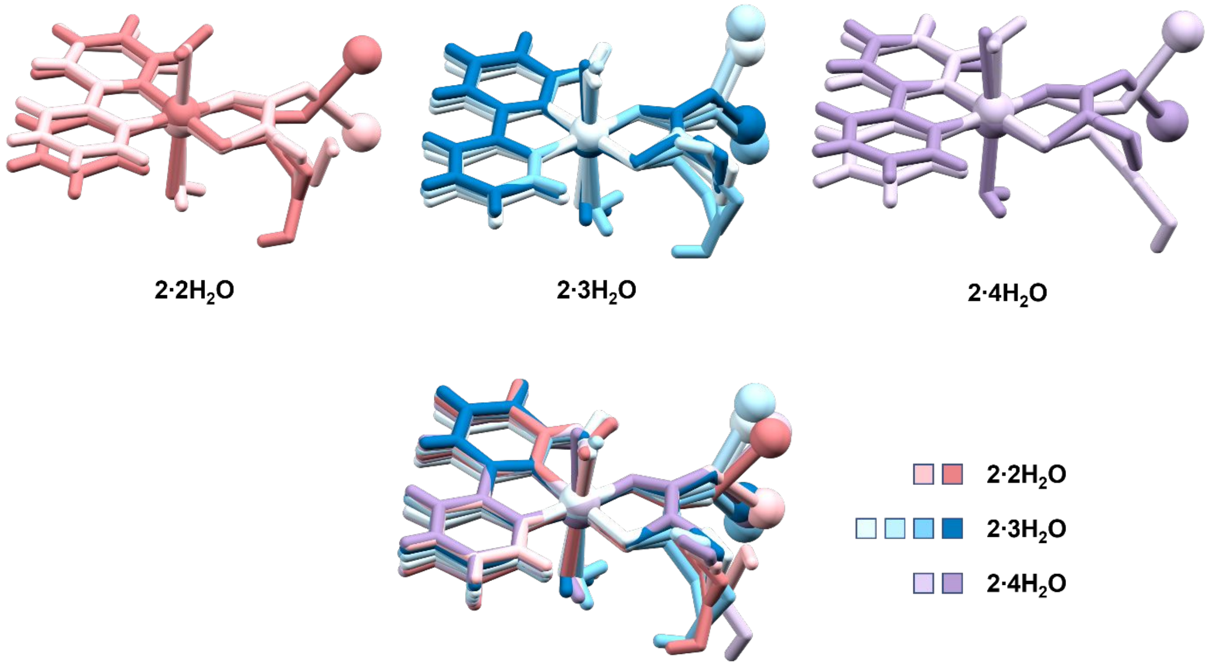


**Figure S4.** Overlay of the complex cations in the following structures: **2·2$H_2O$**, **2·3$H_2O$**, and **2·4$H_2O$**, over copper atoms and coordinated atoms in the equatorial plane. The water molecules of crystallization, sulfate anions, and hydrogen atoms from L-homoserinato side chains (except hydroxyl hydrogen atoms) have been omitted for clarity.

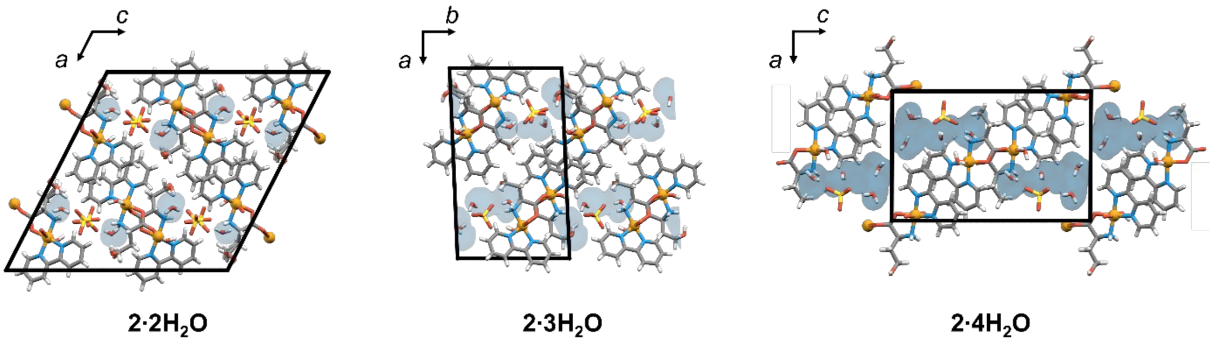


**Figure S5.** Crystal packing in **2·2$H_2O$, 2·3$H_2O$** and **2·4$H_2O$**. Blue color represents the surface around pockets of crystallization water molecules.

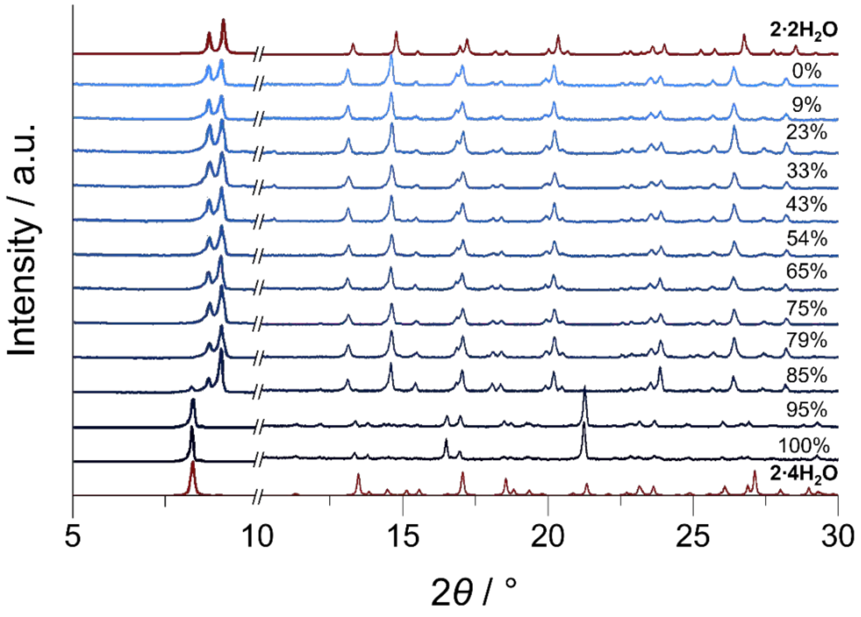


**Figure S6.** Powder X-ray diffraction (PXRD) patterns of the sample of **2·2$H_2O$** aged in atmospheres of different relative humidities at 20 °C for 10 days. PXRD patterns calculated from single-crystal structure data of **2·2$H_2O$** and **2·4$H_2O$** are shown in red, while experimental PXRD patterns are displayed in shades of blue. Parts of the diffraction patterns separated by broken lines are not on the same intensity scale.

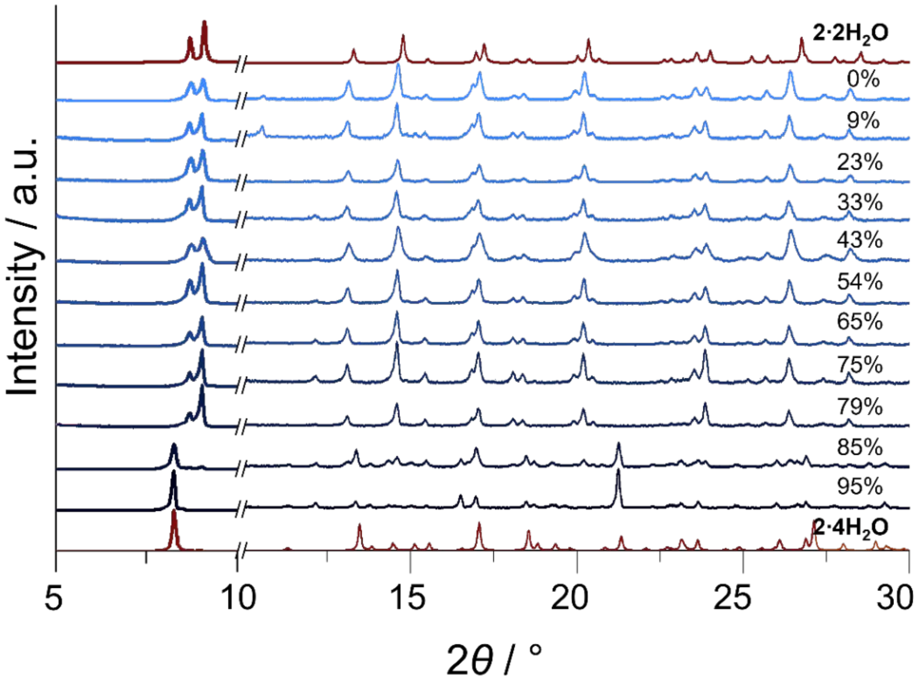


**Figure S7.** Powder X-ray diffraction (PXRD) patterns of the sample of **2·2$H_2O$** aged in atmospheres of different relative humidities at 20 °C for 60 days. PXRD patterns calculated from the single-crystal structure of **2·2$H_2O$** and **2·4$H_2O$** are shown in red, while experimental PXRD patterns are displayed in shades of blue. Parts of the diffraction patterns separated by broken lines are not on the same intensity scale.

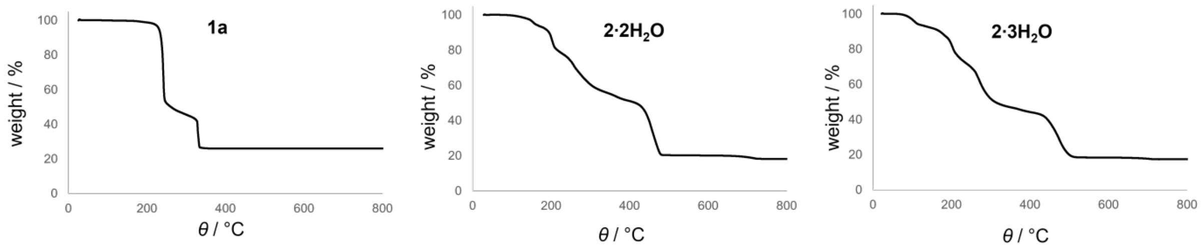


**Figure S8.** TGA curves of **1a**, **2·2H$_2$O** and **2·3H$_2$O**.

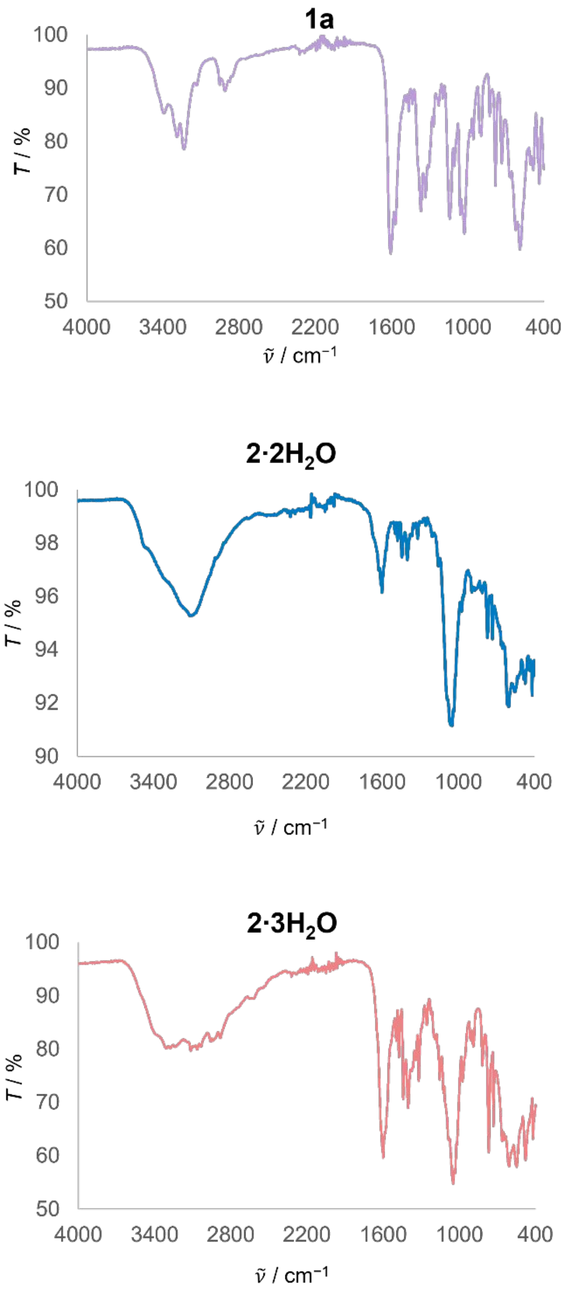


**Figure S9.** IR(ATR) spectra of **1a**, **2·2H$_2$O** and **2·3H$_2$O** in solid state.

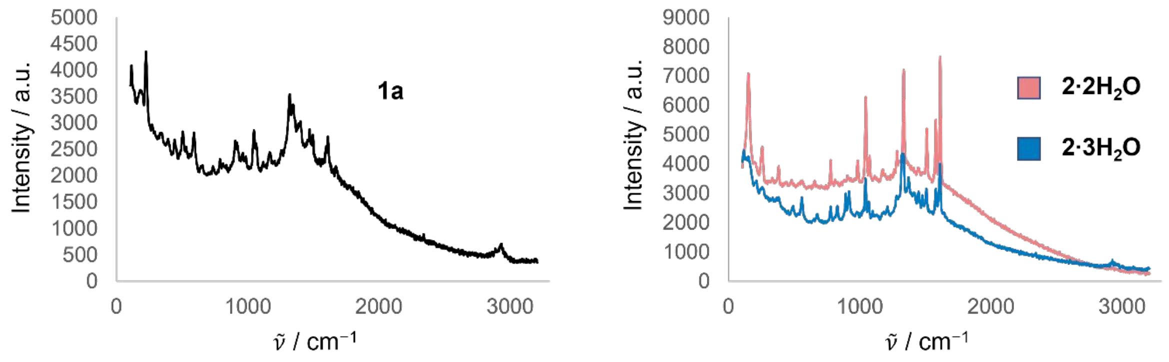


**Figure S10.** Raman spectra of **1a**, **2·2$H_2O$** and **2·3$H_2O$** in solid state.

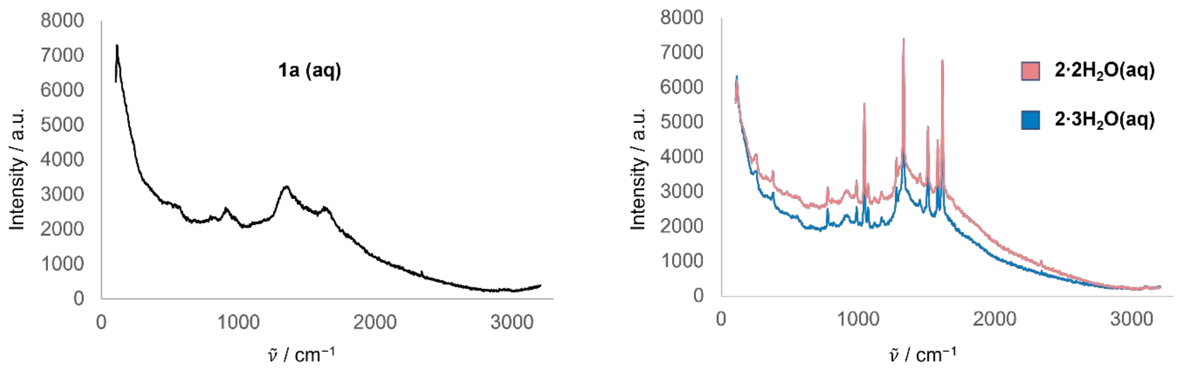


**Figure S11.** Raman spectra of **1a**, **2·2$H_2O$** and **2·3$H_2O$** in aqueous solution ($c$ = 0.1 mol $L^{-1}$).

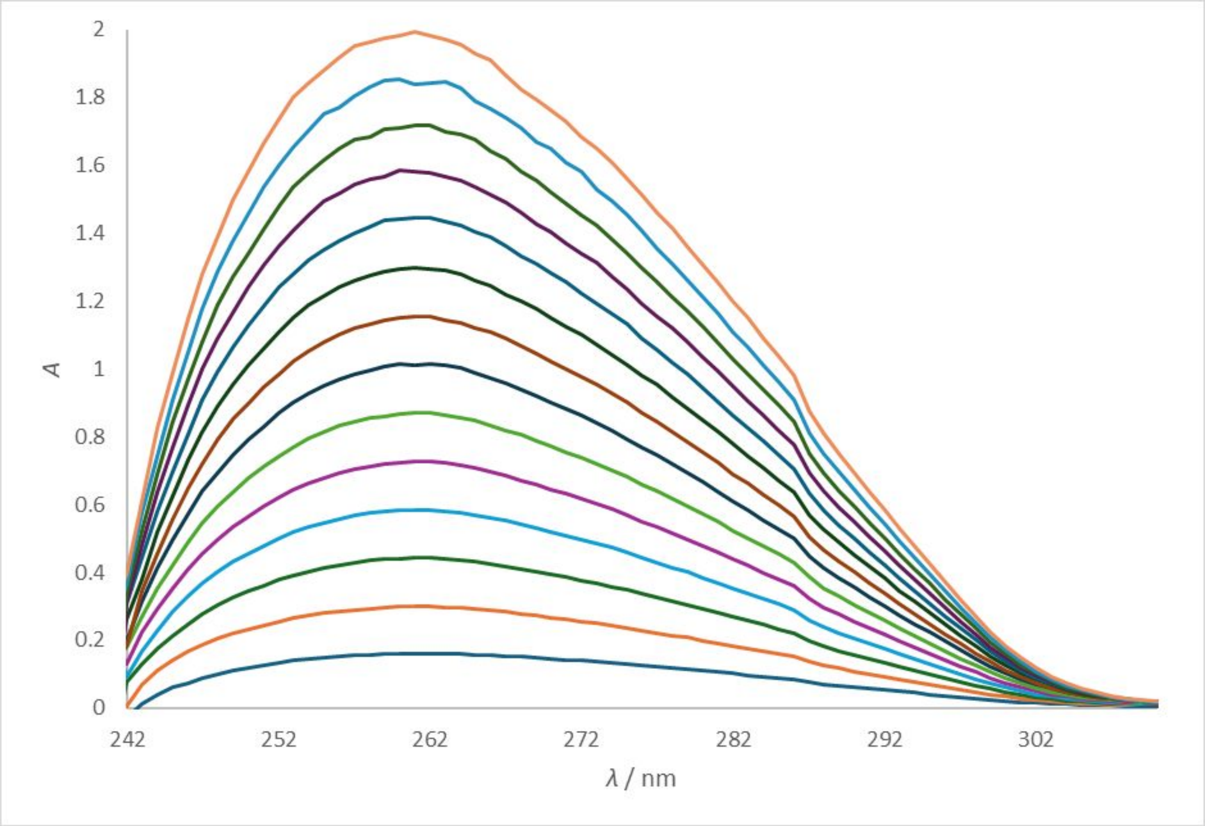


**Figure S12.** The DNA concentration was determined by measuring the UV absorption at 260 nm, taking the molar absorption coefficient ($\varepsilon_{260}$) of **ds(CGCGAATTCGCG)** as 199349 L mol$^{-1}$ cm$^{-1}$.

**Table S1.** Distances ([Å]) within the polyhedra of copper coordination spheres in the crystal structures of **1a, 1b·$H_2O$**, **2·2$H_2O$, 2·3$H_2O$** and **2·4$H_2O$**

**Bond lengths [Å]**

| | **1a** | **1b·$H_2O$** | **2·2$H_2O$** | **2·3$H_2O$** | **2·4$H_2O$** |
|---|---|---|---|---|---|
| Cu1-O11 | 1.9657(17) | 1.961(4) | 1.945(4) | 1.940(13) | 1.955(4) |
| Cu1-O21 | 1.9410(17) | 1.961(4) | - | - | - |
| Cu1-N1 | 1.976(2) | 1.977(6) | 1.993(5) | 1.970(14) | 1.988(4) |
| Cu1-N2 | 1.988(2) | 1.977(6) | - | - | - |
| Cu1-O1D | 2.394(2)[a] | 2.664(5) | - | - | - |
| Cu1-O2D | - | 2.690(5) | - | - | - |
| Cu1-N11 | - | - | 1.989(5) | 1.983(13) | 1.979(5) |
| Cu1-N110 | - | - | 2.002(4) | 1.988(12) | 2.008(4) |
| Cu1-O12 | - | - | 2.796(4) | 2.680(11)* | 2.764(5)[b] |
| Cu1-O13 | - | - | 2.478(5) | 2.403(11) | 2.355(5) |
| Cu2-O21 | - | - | 1.943(4) | 1.962(11) | 1.953(4) |
| Cu2-O22 | - | - | 2.701(4) | 2.680(11) | 2.804(5) |
| Cu2-O23 | - | - | 2.436(5) | 2.559(11) | 2.426(5) |
| Cu2-N2 | - | - | 1.989(5) | 2.022(13) | 2.001(4) |
| Cu2-N21 | - | - | 1.992(5) | 2.009(16) | 2.000(4) |
| Cu2-N210 | - | - | 1.991(4) | 1.998(12) | 1.999(4) |
| Cu3-O31 | - | - | | 1.926(12) | - |
| Cu3-O33 | - | - | | 2.631(11) | - |
| Cu3-O42 | - | - | | 2.511(11) | - |
| Cu3-N3A | - | - | | 1.964(14) | - |
| Cu3-N3B | - | - | | 1.964(14) | - |
| Cu3-N31 | - | - | | 1.982(13) | - |
| Cu3-N310 | - | - | | 1.988(12) | - |
| Cu4-O41 | - | - | | 1.961(12) | - |
| Cu4-O43 | - | - | | 2.386(11) | - |
| Cu4-O32 | - | - | | 2.782(12)* | - |

| | | | |
|---|---|---|---|
| Cu4-N4 | - | 2.006(13) | - |
| Cu4-N41 | - | 1.995(13) | - |
| Cu4-N410 | - | 2.021(12) | - |

**Table S2.** Valence bond angles ([°]) within the distorted octahedral copper coordination spheres in the crystal structures of **1b·$H_2O$**, **2·2$H_2O$**, **2·3$H_2O$** and **2·4$H_2O$**

| **1b·$H_2O$** | **Bond angles around Cu1** | | | |
|---|---|---|---|---|
| | O11–Cu1–O21 98.03(19) | | | |
| | O11–Cu1–N1 83.6(2) | | | |
| | O11–Cu1–N2 173.5(2) | | | |
| | O1D –Cu1–O11 86.78(19) | | | |
| | O2D –Cu1–O11 86.40(19) | | | |
| | O21–Cu1–N1 174.5(2) | | | |
| | O21–Cu1–N2 84.0(2) | | | |
| | O1D–Cu1–O21 87.02(16) | | | |
| | O2D–Cu1–O21 86.29(17) | | | |
| | N1–Cu1–N2 95.0(2) | | | |
| | O1D–Cu1–N1 87.8(2) | | | |
| | O2D–Cu1–N1 99.1(2) | | | |
| | O1D–Cu1–N2 99.5(2) | | | |
| | O2D–Cu1–N2 87.6(2) | | | |
| | O1D–Cu1–O2D 169.69(16) | | | |
| **2·2$H_2O$** | **Bond angles around Cu1** | **Bond angles around Cu2** | | |
| | O11–Cu1–O13 92.69(17) | O22–Cu2–N210 79.39(17) | | |
| | O11–Cu1–N1 84.60(19) | O21–Cu2–O23 97.73(19) | | |
| | O11–Cu1–N11 93.11(18) | O21–Cu2–N2 84.77(18) | | |
| | O11–Cu1–N110 168.0(2) | O21–Cu2–N21 93.02(19) | | |
| | O11–Cu1–O12 89.68(16) | O21–Cu2–N210 168.3(2) | | |
| | O13–Cu1–N1 89.5(2) | O21–Cu2–O22 90.38(18) | | |
| | O13–Cu1–N11 92.0(2) | O23–Cu2–N2 90.0(2) | | |
| | O13–Cu1–N110 98.14(17) | O23–Cu2–N21 94.2(2) | | |
| | O12–Cu1–O13 171.98(15) | O23–Cu2–N210 93.22(18) | | |
| | N1–Cu1–N11 177.36(16) | O22–Cu2–O23 168.78(14) | | |
| | N1–Cu1–N110 100.7(2) | N2–Cu2–N21 175.4(2) | | |
| | O12–Cu1–N1 83.1(2) | N2–Cu2–N210 99.53(19) | | |
| | N11–Cu1–N110 81.34(19) | O22–Cu2–N2 82.97(19) | | |
| | O12–Cu1–N11 95.56(19) | N21–Cu2–N210 81.90(19) | | |

| | | | | |
|---|---|---|---|---|
| | O12−Cu1−N110 80.30(16) | O22−Cu2−N21 93.07(19) | | |
| **2·3$H_2$O** | **Bond angles around Cu1** | **Bond angles around Cu2** | **Bond angles around Cu3** | **Bond angles around Cu4** |
| | O13−Cu1−O22 173.1(4) | O23−Cu2−N21 95.1(5) | O31−Cu3−O33 89.7(4) | O41−Cu4−O43 96.0(4) |
| | N1−Cu1−N11 174.6(6) | O23−Cu2−N210 90.5(4) | O31−Cu3−O42 91.6(4) | O41−Cu4−N4 84.1(5) |
| | N1−Cu1−N110 99.2(5) | N2−Cu2−N21 175.7(6) | O31−Cu3−N3A 83.9(5) | O41−Cu4−N41 94.1(5) |
| | O22−Cu1−N1 91.0(5) | N2−Cu2−N210 102.0(5) | O31−Cu3−N31 93.2(5) | O41−Cu4−N410 171.4(5) |
| | N11−Cu1−N110 82.0(5) | N21−Cu2−N210 81.6(5) | O31−Cu3−N310 173.4(6) | O32−Cu4−O41 90.1(4) |
| | O22−Cu1−N11 83.8(4) | O21−Cu2−O23 91.2(4) | O33−Cu3−O42 170.5(5) | O43−Cu4−N4 93.5(5) |
| | O22−Cu1−N110 82.7(4) | O21−Cu2−N2 83.6(5) | O33−Cu3−N3A 84.0(5) | O43−Cu4−N41 94.4(5) |
| | O11−Cu1−O13 98.5(5) | O12−Cu2−O21 91.1(4) | O33−Cu3−N31 95.2(4) | O43−Cu4−N410 91.2(4) |
| | O11−Cu1−N1 84.4(6) | O12−Cu2−O23 173.6(4) | O33−Cu3−N310 93.7(4) | O32−Cu4−O43 173.9(4) |
| | O11−Cu1−N11 93.6(5) | O12−Cu2−N2 87.0(5) | O42−Cu3−N3A 86.7(5) | N4−Cu4−N41 172.1(6) |
| | O11−Cu1−N110 170.3(6) | O12−Cu2−N21 90.8(5) | O42−Cu3−N31 94.2(4) | N4−Cu4−N410 100.1(5) |
| | O11−Cu1−O22 88.3(4) | O12−Cu2−N210 87.8(4) | O42−Cu3−N310 86.0(4) | O32−Cu4−N4 87.8(5) |
| | O13−Cu1−N1 90.8(5) | O21−Cu2−N210 174.2(6) | N3A−Cu3−N31 177.0(6) | N41−Cu4−N410 80.7(5) |
| | O13−Cu1−N11 94.5(5) | O23−Cu2−N2 87.3(5) | N3A−Cu3−N310 102.0(5) | O32−Cu4−N41 84.5(4) |
| | O13−Cu1−N110 90.4(4) | O21−Cu2−N21 92.7(5) | N31−Cu3−N310 80.9(5) | O32−Cu4−N410 82.6(4) |
| **2·4$H_2$O** | **Bond angles around Cu1** | **Bond angles around Cu2** | | |
| | O11−Cu1−O13 96.08(17) | N2−Cu2−N21 175.46(17) | | |
| | O11−Cu1−N1 83.98(16) | N2−Cu2−N210 103.4(2) | | |
| | O11−Cu1−N11 92.92(17) | O22−Cu2−N2 95.65(19) | | |
| | O11−Cu1−N110 167.8(2) | N21−Cu2−N210 81.01(19) | | |
| | O11−Cu1−O12 84.99(16) | O22−Cu2−N21 84.25(18 | | |
| | O13−Cu1−N1 95.33(18) | O22−Cu2−N210 78.94(17) | | |
| | O13−Cu1−N11 91.17(18) | O21−Cu2−O23 99.63(19) | | |
| | O13−Cu1−N110 94.88(17) | O21−Cu2−N2 83.10(17) | | |
| | O12−Cu1−O13 176.34(13) | O21−Cu2−N21 92.37(17) | | |
| | N1−Cu1−N11 173.1(2) | O21−Cu2−N210 167.5(2) | | |
| | N1−Cu1−N110 100.33(19) | O21−Cu2−O22 89.90(18) | | |
| | O12−Cu1−N1 88.27(18) | O23−Cu2−N2 88.0(2) | | |
| | N11−Cu1−N110 81.50(19) | O23−Cu2−N21 92.8(2) | | |
| | O12−Cu1−N11 85.27(18) | O23−Cu2−N210 91.33(18) | | |

| | | | | |
|---|---|---|---|---|
| | O12−Cu1−N110 83.75(16) | O22−Cu2−O23 170.16(13) | | |

**Table S3.** Selected hydrogen bonds for compounds **1a**, **1b·$H_2O$, 2·2$H_2O$, 2·3$H_2O$** and **2·4$H_2O$.**

| Compound | D–H···A | *d*(D–H···A) / Å | ∠( D–H···A) / ° |
|---|---|---|---|
| **1a** | N1–H1B···O12[a] | 2.899(3) | 162 |
| | N1–H1C···O2D[b] | 2.899(2) | 167 |
| | O1D–H1D···O11[c] | 2.855(3) | 170(3) |
| | N2–H2B···O12[d] | 2.922(3) | 170 |
| | N2–H2C···O22[b] | 2.994(2) | 176 |
| | O2D–H2D···O21[e] | 3.140(3) | 133(4) |
| | O2D–H2D···O22[e] | 2.812(3) | 163(4) |
| **1b·$H_2O$** | N1–H1B···O12[c] | 3.241(8) | 139 |
| | N1–H1C···O1D | 2.902(8) | 102 |
| | N1–H1C···O21[f] | 3.104(7) | 168 |
| | O1D–H1D···O12[g] | 2.707(7) | 165(7) |
| | N2–H2B···O22[f] | 3.268(8) | 139 |
| | N2–H2C···O2D | 2.889(8) | 101 |
| | N2–H2C···O11[c] | 3.086(7) | 167 |
| | O2D–H2D···O22[g] | 2.715(7) | 160(9) |
| **2·2$H_2O$** | O13–H13A···O13S | 2.749(11) | 163(8) |
| | O13H–13A···O17S | 2.62(3) | 155(9) |
| | O13–H13B···O12[h] | 2.871(6) | 170(13) |
| | N1–H1B···O13S | 3.080(9) | 164 |
| | N1–H1B···O15S | 2.80(3) | 146 |
| | N1–H1A···O2W[i] | 2.969(9) | 152 |
| | N2–H2A···O11S | 2.848(8) | 161 |
| | N2–H2A···O18S | 3.01(3) | 160 |
| | N2–2H2B···O1W[j] | 3.095(9) | 171 |
| | O1D–1H1D1···O2W | 2.906(12) | 166 |
| | O2D2–H2D2···O11S[i] | 2.944(12) | 142 |
| **2·3$H_2O$** | O13–H13A···O22 | 2.841(13) | 160 |
| | O13–H13B···O14S | 2.664(14) | 154 |

| | | | |
|---|---|---|---|
| | O23–H23A···O12[e] | 2.870(13) | 164 |
| | O33–H33A···O5W[i] | 2.690(14) | 140 |
| | O33–H33B···O42[c] | 2.836(13) | 127 |
| | O43–H43A···O21S | 2.721(13) | 159 |
| | O43–H43B···O32 | 2.792(13) | 165 |
| | N1–H1B···O13S | 3.197(17) | 153 |
| | N1–H1C···O3W | 2.892(15) | 176 |
| | N2–H2B···O11S[j] | 3.071(17) | 173 |
| | N2–H2C···O11 | 3.359(15) | 132 |
| | N3B–H3B3···O41 | 3.290(15) | 117 |
| | N3A–H3A2···O25S[i] | 3.062(16) | 133 |
| | N4–H4C···O27S | 3.088(17) | 163 |
| | N4–H4C···O23S | 2.964(17) | 127 |
| | N4–H4B···O4W[e] | 2.959(16) | 144 |
| | O1D–H1D···O13S | 2.721(19) | 140 |
| | O2D–H2D···O11S[j] | 2.770(19) | 135 |
| | O3D1–H3D1···O25S[j] | 2.81(3) | 163 |
| | O4D–H4D···O4W[e] | 2.841(19) | 160 |
| **2·4$H_2O$** | O13–H13A···O12S[j] | 2.678(5) | 163(6) |
| | O13–H13B···O12[k] | 2.840(6) | 171(4) |
| | O23–H23B···O22[d] | 2.850(7) | 169(6) |
| | O23–H23A···O1W[l] | 2.865(7) | 148(7) |
| | N1–1H1A···O11S[e] | 2.936(6) | 160 |
| | N1–H1B···O2W[m] | 3.129(8) | 177 |
| | N2–H2A···O14S[i] | 2.937(6) | 165 |
| | N2–2H2B···O3W[l] | 3.081(8) | 154 |
| | O1D–H1D···O4W | 2.638(10) | 135 |
| | O2D–H2D···O14S[i] | 2.786(5) | 126 |

[a] -x,-1/2+y,1-z; [b] 1-x,1/2+y,2-z; [c] -1+x,y,z; [d] 1-x,-1/2+y,1-z; [e]1+x,y,z; [f] x,y,-1+z; [g] -1+x,y,-1+z; [h] 3/2-x,1/2+y,2-z; [i]x,-1+y,z; [j] x,1+y,z; [k] 2-x,1/2+y,-z; [l] x,-2+y,z; [m] 1-x,-1/2+y,-z

**Table S4.** Electronic absorption bands of Cu(II) compounds in 10 mmol $L^{-1}$ Tris-base buffer (pH 7.4) at room temperature. UV spectra were measured at 32 $\mu$mol $L^{-1}$ for **1a** and **2·2H$_2$O,** and at 16 $\mu$mol $L^{-1}$ for **2·3H$_2$O**. Visible spectra were measured at 2 mmol L $^{-1}$, 4 mmol $L^{-1}$ and 1 mmol $L^{-1}$ for **1a**, **2·2H$_2$O** and **2·3H$_2$O**, respectively.

| Cu(II) compounds | d-d band $\lambda_{max}$ / nm | $\varepsilon$ / $M^{-1}$ $cm^{-1}$ | CT[a] bands $\lambda_{max}$ / nm | $\varepsilon$ / $M^{-1}$ $cm^{-1}$ |
|---|---|---|---|---|
| **1a** | 628 | 66 | 263 | 3314 |
| **2·2H$_2$O** | 607 | 82 | 256<br>304<br>316 | 11989<br>12851<br>13430 |
| **2·3H$_2$O** | 609 | 73 | 258<br>304<br>317 | 11238<br>9364<br>11210 |

[a]CT- charge transfer

**Table S5.** Crystallographic data for compounds **1a, 1b·$H_2O$, 2·2$H_2O$, 2·3$H_2O$** and **2·4$H_2O$**

| | **1a** | **1b·$H_2O$** | **2·2$H_2O$** | **2·3$H_2O$** | **2·4$H_2O$** |
|---|---|---|---|---|---|
| Formula | $C_8H_{16}CuN_2O_6$ | $C_8H_{18}CuN_2O_7$ | $C_{28}H_{40}Cu_2N_6O_{14}S$ | $C_{28}H_{42}Cu_2N_6O_{15}S$ | $C_{28}H_{44}Cu_2N_6O_{16}S$ |
| Formula weight [g mol$^{-1}$] | 299.77 | 317.78 | 843.80 | 861.81 | 879.85 |
| $\lambda$ [Å] | 0.71073 | 1.54184 | sync, 0.70000 | 1.54184 | 0.71073 |
| Crystal system | monoclinic | monoclinic | monoclinic | triclinic | monoclinic |
| Space group | $P2_1$ | $P2_1$ | $C2$ | $P1$ | $P2_1$ |
| $a$ [Å] | 7.7516(2) | 5.6593(1) | 22.860(1) | 6.9782(2) | 12.7941(5) |
| $b$ [Å] | 8.4814(2) | 18.1943(2) | 7.050(1) | 12.0823(2) | 6.9697(3) |
| $c$ [Å] | 8.4194(2) | 5.6687(1) | 22.810(1) | 20.4638(2) | 19.7215(7) |
| $\alpha$ [°] | 90 | 90 | 90 | 87.595(1) | 90 |
| $\beta$ [°] | 98.318(3) | 93.732(1) | 117.120(4) | 89.926(1) | 90.281(4) |
| $\gamma$ [°] | 90 | 90 | 90 | 87.188(2) | 90 |
| $V$ [Å$^3$] | 547.71(2) | 582.451(16) | 3272.0(5) | 1721.76(6) | 1758.57(12) |
| $Z$ | 2 | 2 | 4 | 2 | 2 |
| $T$ [K] | 150 | 170 | 100 | 170 | 150 |
| $\rho$ [g cm$^{-3}$] | 1.818 | 1.812 | 1.713 | 1.662 | 1.662 |
| $\mu$ [mm$^{-1}$] | 2.014 | 2.979 | 1.320 | 2.794 | 1.351 |
| $\theta$ range [°] | 4.6, 30.0 | 4.9, 74.9 | 1.8, 28.3 | 3.7, 65.1 | 4.3, 32.8 |
| Obs. reflections ($I$ > 2 $\sigma$ ($I$)) | 2585 | 2384 | 7921 | 10804 | 5273 |
| Number of parameters | 162 | 175 | 535 | 923 | 516 |
| $R_1$ (observed reflections)$^1$ | 0.0190 | 0.0454 | 0.0534 | 0.0626 | 0.0543 |
| $wR_2$ (all data)$^2$ | 0.0469 | 0.1169 | 0.1387 | 0.1731 | 0.1094 |
| $S^3$ | 1.10 | 1.12 | 1.08 | 1.04 | 0.99 |
| min/max residual electron density [e Å$^{-3}$] | -0.28, 0.26 | -0.79, 1.22 | -1.45, 0.88 | -0.80, 1.07 | -0.83, 0.93 |
| Flack parameter | 0.011(6) | 0.05(6) | 0.029(5) | 0.17(4) | -0.003(17) |
| CCDC no. | 2465082 | 2465083 | 2465085 | 2465084 | 2465086 |

## Proliferation assays, MTT test

The panel cell lines were inoculated in parallel onto a series of standard 96-well microtiter plates on day 0, at 1×10$^4$ to 3×10$^4$ cells/mL, depending on the doubling times of specific cell line. Test agents were then added in five 10-fold dilutions (10$^{-8}$ to 10$^{-4}$ M) and incubated for a further 72 hours. Working dilutions were freshly prepared on the day of testing. After 72 hours of incubation the cell growth rate was evaluated by performing the MTT assay, which detects dehydrogenase activity in viable cells. The

percentage of growth (PG) of the cell lines was calculated according to one or the other of the following two expressions:

If (Atest –Atzero) ≥ 0 then:

PG = 100 × (Atest – Atzero) / (Acont-Atzero)

If (Atest – Atzero) < 0 then:

PG = 100 × (Atest – Atzero) / Atzero)

Where:

Atzero = the average absorbance before exposure of cells to the test compound

Atest = the average absorbance after the desired period of time (72 h)

Acont = the average absorbance after 72 hours with no exposure of cells to the test compound

Each test point was performed in quadruplicate in minimum two individual experiments. The results are expressed as IC50, a concentration necessary for 50% of inhibition. The $IC_{50}$ values for each compound are calculated from dose-response curves using linear regression analysis by fitting the test concentrations that give PG values above and below the respective reference value (e.g. 50 for $IC_{50}$). Therefore, a "real" value for any of the response parameters is obtained only if at least one of the tested drug concentrations falls above, and likewise at least one falls below the respective reference value. If however, for a given cell line all of the tested concentrations produce PGs exceeding the respective reference level of effect (e.g. PG value of 50), then the highest tested concentration is assigned as the default value. In the screening data report, that default value is preceded by a ">" sign.

**Table S6.** Absorption titration measurements were done by varying the concentration of **ds(CGCGAATTCGCG)** while keeping the concentration of **2·2$H_2O$** constant (30 mmol $L^{-1}$) in 10 mmol $L^{-1}$ Tris-base buffer (pH 7.4) at room temperature.

| *c*(**2·2$H_2O$**) / μM | *c*(**dsDNA**) / μM | $V_{final}$ / μL | $c_0$(**dsDNA**)/ μM | $V_1$(dsDNA) / μL |
|---|---|---|---|---|
| 30 | 0 | 500 | 250 | 0 |
| | 0.5 | 501 | | 1 |
| | 1 | 502 | | 2 |
| | 1.5 | 503 | | 3 |
| | 2 | 504 | | 4 |
| | 2.5 | 505 | | 5 |
